# Adhesion of retinal cells to gold surfaces by biomimetic molecules

**DOI:** 10.3389/fcell.2024.1438716

**Published:** 2024-08-28

**Authors:** Gal Shpun, Amos Markus, Nairouz Farah, Zeev Zalevsky, Yossi Mandel

**Affiliations:** ^1^ The Alexander Kofkin Faculty of Engineering, Bar Ilan University, Ramat Gan, Israel; ^2^ School of Optometry and Visual Science, Faculty of Life Sciences, Bar Ilan University, Ramat Gan, Israel; ^3^ Bar Ilan Institute for Nanotechnology and Advanced Materials (BINA), Bar Ilan University, Ramat Gan, Israel; ^4^ The Gonda Multidisciplinary Brain Research Centre, Bar-Ilan University, Ramat Gan, Israel

**Keywords:** cell-adhesion, biomimetics, RGD, YIGSR, neural electrode interface, regenerative medicine, retinal prostheses, tissue engineering

## Abstract

**Background:**

Neural cell-electrode coupling is crucial for effective neural and retinal prostheses. Enhancing this coupling can be achieved through surface modification and geometrical design to increase neuron-electrode proximity. In the current research, we focused on designing and studying various biomolecules as a method to elicit neural cell-electrode adhesion via cell-specific integrin mechanisms.

**Methods:**

We designed extracellular matrix biomimetic molecules with different head sequences (RGD or YIGSR), structures (linear or cyclic), and spacer lengths (short or long). These molecules, anchored by a thiol (SH) group, were deposited onto gold surfaces at various concentrations. We assessed the modifications using contact angle measurements, fluorescence imaging, and X-ray Photoelectron Spectroscopy (XPS). We then analyzed the adhesion of retinal cells and HEK293 cells to the modified surfaces by measuring cell density, surface area, and focal adhesion spots, and examined changes in adhesion-related gene and integrin expression.

**Results:**

Results showed that YIGSR biomolecules significantly enhanced retinal cell adhesion, regardless of spacer length. For HEK293 cells, RGD biomolecules were more effective, especially with cyclic RGD and long spacers. Both cell types showed increased expression of specific adhesion integrins and proteins like vinculin and PTK2; these results were in agreement with the adhesion studies, confirming the cell-specific interactions with modified surfaces.

**Conclusion:**

This study highlights the importance of tailored biomolecules for improving neural cell adhesion to electrodes. By customizing biomolecules to foster specific and effective interactions with adhesion integrins, our study provides valuable insights for enhancing the integration and functionality of retinal prostheses and other neural implants.

## 1 Introduction

Cell and neuronal adhesion to surfaces plays a pivotal role in various biomedical applications (Dahmen et al., 2004), particularly in the fields of tissue engineering (Kasemo, 2002; Roach et al., 2010; Khalili and Ahmad, 2015; Farrukh et al., 2018; Shin et al., 2003) and electrode interfaces (Shoffstall and Capadona, 2018; Blau, 2013; Woeppel et al., 2017) in neuroprostheses (Yu et al., 2008; Szostak et al., 2017). One critical aspect of cell adhesion is the proximity of cells to electrode surfaces (Yu et al., 2008), since it affects the cell-electrode electrical coupling and the electric field distribution. Thus, achieving a precise and controlled cell-electrode interaction is essential for optimizing signal recordings or neural stimulation in various neural prostheses in general, and in electronic retinal implants, in particular (Woeppel et al., 2017).

The methods employed to promote cell adhesion to surfaces and electrodes encompass a broad range of strategies that leverage both biological and physical factors to create surfaces that are biocompatible and encourage cell attachment (Roach et al., 2010; Farrukh et al., 2018). One approach is to use electrostatic forces, which utilize the negative charge of the cell membrane, as with the poly-L-lysine (PLL), poly-D-lysine (PDL), and polyethylene mine (PEI) coatings (Yu et al., 2008). Another strategy is to create topographical cues such as grooves and patterned substrates, which create textured surfaces that enhance cell attachment (Yu et al., 2008). Yet another approach is treatment with wet or dry plasma; both of which increase the surface wettability and activate the surface with reactive groups (Wertheimer, 2015; Oehr, 2003).

To enable a more cell-specific approach, one can use the advantage of cell-substrate interactions mediated through integrins, a superfamily of transmembrane cell surface receptors that play a central role in dynamic mechanotransduction signalling with the extracellular matrix (ECM) (Barczyk et al., 2010; Takada et al., 2007). In humans, there are 18 different α subunits and 8 β subunits of integrins, leading to the potential formation of numerous integrin heterodimers, each with unique ligand-binding specificities and functions (Barczyk et al., 2010; Takada et al., 2007; Hynes, 2002). The α and β subunits possess large extracellular domains that are responsible for ECM ligand recognition and binding, a single transmembrane domain, and short cytoplasmic tails that interact with intracellular proteins (such as Talin, Vinculin, and Pixelin) to trigger signalling cascades (Takada et al., 2007; Xiong et al., 2001; Perdih and Sollner Dolenc, 2010). Previous studies have shown that β_1_ and β_3_ subunits, particularly through the α_5_β_1_, α_V_β_3_, and α_IIb_β_3_ complexes, play pivotal roles in promoting strong cell adhesion (Perdih and Sollner Dolenc, 2010; Mas-Moruno et al., 2016; Hersel et al., 2003), including the formation of focal adhesion complexes, with α_IIb_β_3_ predominantly responsible for platelet coagulation (Perdih and Sollner Dolenc, 2010; Mitsios et al., 2010).

In recent years, there has been much interest in using peptidomimetics, i.e., short ECM-like peptides, to enhance the adhesion of specific cells to surfaces. These peptidomimetics imitate the cell microenvironment and interact with the cellular integrins by presenting the minimal ECM recognition motifs, therefore leading to cell adhesion (Farrukh et al., 2018; Ruoslahti and Pierschbacher, 1987; Ludwig et al., 2021; Chen et al., 2013). Among the major advantages of these biomolecules is the ability to control and pattern attachment to surfaces that do not trigger immune responses and are therefore less susceptible to enzymatic degradation (Perdih and Sollner Dolenc, 2010; Hersel et al., 2003).

The basic structure of these biomolecules consists of three parts, namely, the anchor, spacer, and reactive head group. The anchor is attached to the surface by a stable covalent bond; its type, density, and orientation significantly affect the linkage strength (Chen et al., 2013; Ranieri et al., 1994; Ranieri et al., 1995; Xue et al., 2014). The spacer links the anchor to the ligand head group, maintaining an optimal distance between the surface and catalytic pocket within the cell receptors (usually the integrins), where its length and flexibility (Lee et al., 2010; Kumai et al., 2016) influence accessibility to cell receptors. Lastly, the head group peptide interacts with the cell surface receptors, primarily integrins, and mimics natural ECM motifs; thus, its specificity and affinity for integrin subtypes is crucial for effective adhesion (Mas-Moruno et al., 2016; Hersel et al., 2003). Important representatives of this peptide family are the Fibronectin motif Arginine-Glycine-Aspartic Acid (RGD) (Hersel et al., 2003; Ritz et al., 2017), the Laminin adhesion motifs Tyrosine-Isoleucine-Glycine-Serine-Arginine (YIGSR) (Farrukh et al., 2018), and Ile-Lys-Val-Ala-Val (IKVAV).

Most research in this field has focused on fibroblasts, mesenchymal, and general neural cells such as PC12 and cortical cells (Woeppel et al., 2017); however, there is a lack of information about the effect of peptidomimetics on retinal cell attachment to surfaces. Similarly, previous studies, aiming to improve the electrode-neural interface for retinal prostheses, focused on the non-cell-specific mechanisms by using the coatings of Laminin, Fibronectin, or PLLA, which have primarily been assessed with non-retinal cells (Thakur et al., 2018; Sikder et al., 2021; Aktas et al., 2024). Only limited studies have addressed this aspect using retinal cells, predominantly in avian models (Huber et al., 1998).

The current work aims to study, for the first time, to the best of our knowledge, the use of short biomolecules to mimic the retinal ECM and to elicit the adhesion of retinal cells to metal electrodes. To this end, we used various molecular designs, in terms of the head group sequence (RGD or YIGSR), its spatial conformation (linear or cyclic), and the spacer length (short or long), as summarized in Table 1. The various biomolecules were attached to gold electrodes via a semi-covalent bond through a thiol group (SH); a cell adhesion assay and a cell spreading surface were used to estimate the efficiency of the biomolecules. Aiming to better understand the specific biomolecule-induced mechanism of cell adhesion, we studied the generation of focal adhesion complex creation and gene regulation, which were associated with various biomolecule surface coatings.

**TABLE 1:** Sequence and conformational structure of the biomolecules used in the study.

Spacer Sequence	Short:(GG)	Long:(Poly-Pro(10))
Linear: RGDfK	CGG-RGDfK	—
Cyclic: c(RGDfK)	CGG-c(RGDfK)	C-PolyPro(10)-c(RGDfK)
YIGSR	CGG-YIGSR	C-PolyPro(10)-YIGSR

## 2 Materials and methods

### 2.1 Cell culture device fabrication

For model electrodes, we used a gold layer deposited on glass. To this end, microscope glass slides (#7105, BOJACK, China) were first cleaned by rinsing in piranha solution (3:1, ammonia solution: hydrogen peroxide), followed by double-distilled water (DDW), and heating to remove moisture in an oven at 120°C for 20 min. Next, Cr/Au (10 nm/100 nm) layers were spatter deposited (Bestec Berlin, Germany) after O_2_+Ar (3 min, 100 W) plasma attaching (Dainer electronics, Pico, Germany) and Ar ion milling (10 s). For the cell density, cell spreading, and focal adhesion experiments, µ-slides [12 wells (#81201, ibidi GmbH, Gräfelfing, Germany)] were mounted on the gold-coated slides and sealed with SYLGARD^®^-184 (#761028-5 EA, Merck, New Jersey, United States), followed by curing at 80°C overnight. Finally, the samples were rinsed twice in DDW, and uprooted by boiling in DDW for 20 min, followed by a 70% ethanol wash and exposure to UV radiation for 30 min in a biological hood. For the gene expression studies, the slides were uprooted without mounting the µ-slides.

### 2.2 Bio-molecule design

#### 2.2.1 Design

The design of the biomolecules used in this study was based on the following: For the anchor, we opted to use the thiol group (SH), which is found in Cysteine amino acid, and spontaneously forms a self-assembly monolayer (SAM) of a semi-covalent bond with gold. Aiming to assess the optimal spacer length, we compared the short amino acid spacer (GG) and the long spacer polyproline (-PPPPPPPPPP), which was shown to be more efficient than the PEG spacer at the same length, regarding rigidity (Pallarola et al., 2017; Pallarola et al., 2014).

Previous studies have shown that the amino acid type and sequence (e.g., RGDxx, where xx represents any amino acid or YIGSR), as well as their conformational structure (e.g., linear, cyclic, or branched), greatly influence the molecule’s affinity to the integrin subtype and especially to the adhesion integrins (Mas-Moruno et al., 2016; Verrier et al., 2002). The RGD motif is a well-known adhesion motif for adhesive basal cells and neurons (Roach et al., 2010; Yu et al., 2008); YIGSR (Rao and Winter, 2009; Ito, 1999) and IKVAV (Yin et al., 2021; Patel et al., 2019; Tashiro et al., 1989) are known to specifically promote the adhesion of neuronal cells.

**FIGURE 1:**
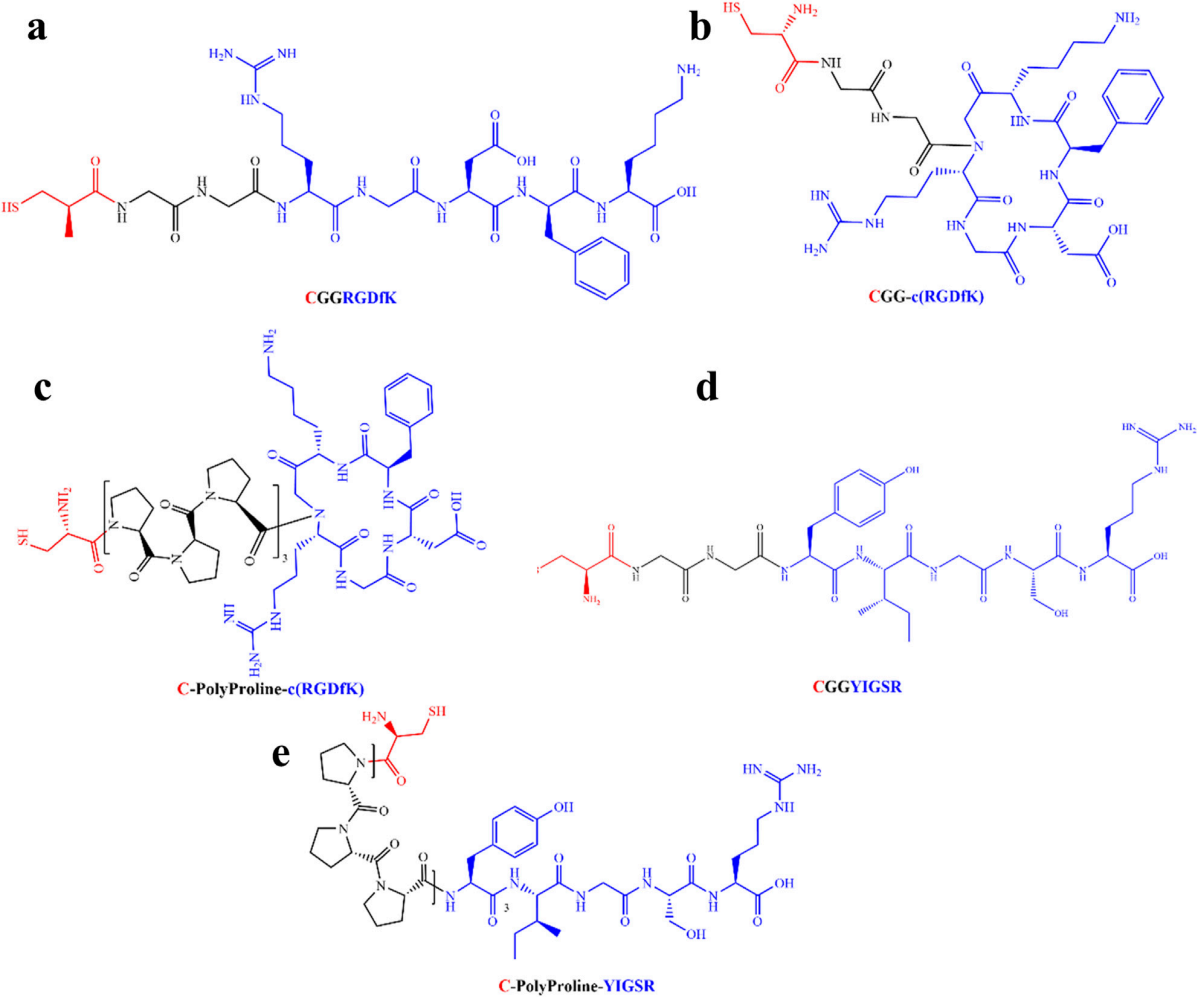
Molecular structure of the biomolecules studied in this study. **(A)** Short linear RGD; Cys-Gly-Gly-Arg-Gly-Asp-D-Phe-Lys (CGGRGDfK). **(B)** Short cyclic RGD; Cys-Gly-Gly-cyclo(Arg-Gly-Asp-D-Phe-Ly), (CGG-c(RGDfK)). **(C)** Long-cyclic RGD; Cys-PolyProline(10)-cyclo(Arg-Gly-Asp-D-Phe-Ly), (C-PolyPro-c(RGDfK)). **(D)** Short YIGSR; Cys-Gly-Gly-Tyr-Ile-Gly-Ser-Arg (CGGYIGSR). **(E)** Long YIGSR; Cys-PolyProline(10)-Tyr-Ile-Gly-Ser-Arg (C-PolyPro-YIGSR). Red–Cys–an anchor to the gold surface, Black–short and long spacers (GG and PolyProline(10), respectively) and blue–the ligand head group (RGDfK and YIGSR).

Taking the above-mentioned considerations into account, we opted to compare the efficiency of linear RGD to cyclic RGD [c (RGD)] and YIGSR with both short and long spacers (see Table 1). Figure 1 presents the types of the investigated biomolecules. As a control, we used DDW-coated surfaces. The molecules were manufactured by the Hanhong Group (Shanghai Hanhong Chemical Co., Ltd., China).

### 2.3 Surface functionalization

#### 2.3.1 Biomolecule coating

The peptides were dissolved in aqueous solution 1:1 (DDW: Acetonitrile (AN), #75058) at a concentration of 1/3 mg/mL and stored until use at −20°C after being divided into several test tubes. Before use, the solutions were thawed and diluted to various concentrations (1/3, 1/6, 1/12, and 1/24 mg/mL). The biomolecule-gold electrode coating was obtained through a self-assembly monolayer (SAM) facilitated by semi-covalent bonds spontaneously forming between the gold and the thiol group (SH) present in the biomolecule, which can be found at cysteine (C) amino acid. Briefly, gold surfaces were coated by immersion for 2 h in the solutions, followed by rinsing with PBS (Khalili and Ahmad, 2015; Shin et al., 2003; Blau, 2013). For control purposes, untreated gold surfaces were soaked only in DDW. All samples were soaked for 2 h at RT and rinsed three times in PBS before cell seeding.

#### 2.3.2 Surface modification analysis

##### 2.3.2.1 X-ray photoelectron spectroscopy (XPS)

To evaluate the assembly of the biomolecules to the gold surface^43 44^, an XPS analysis was performed (Supplementary Figure S1). Survey and high-resolution spectra were acquired at a pass energy of 80 eV and 40 eV, respectively. The source power was set to either 75 W or 150 W. The binding energies of all elements were recalibrated by setting the CC/CH component of the C1 peak to 285 eV. Quantitative surface chemical analysis was performed using high-resolution core-level spectra after removing the nonlinear Shirley background. The measurements were carried out under UHV conditions, at a base pressure of 5 × 10 torr (and no higher than 3 × 10-9torr). Examinations were performed on gold-coated mica glass disks (Electron Microscopy Sciences, Hatfield, PA, United States).

##### 2.3.2.2 Contact angle measurement

To evaluate the gold surface modification by the biomolecules, the contact angle between a water droplet and the surface was measured using a Contact Angle Goniometer (System OCA, model OCA20, Data Physics Instruments GmbH, Filderstadt, Germany). Briefly, gold-coated (100 nm) cover glasses were coated by 2 h of immersion in Acetonitrile-DDW (1:1) solutions for each biomolecule at various concentrations. A drop of 5 μL of DDW was placed in the center of each sample. The measurements (N = 3) were performed at 25°C and 55% moisture; Laplace-Young curve fitting was used to determine the static water contact angle values (Yoon and Mofrad, 2011).

##### 2.3.2.3 Fluorescence microscopy (STED)

Aiming to assess the specific binding of the biomolecules to the gold surface, a chess-like pattern with glass-gold squares (300 nm width, 100 nm height) was immersed for 2 h in a 0.1 mM solution of a fluorescent Cys-RGD-NBD (Nitrobenzofurazan, Ex/Em 467/539 nm), which was a generous gift from Rahimipour’s Lab. Following a triple rinse in DDW, the sample was examined using a Leica TCS SP8 STED microscope (Leica-microsystems, Germany) and compared to a non-coated sample.

### 2.4 Cell culture

#### 2.4.1 Dissociation of rat retinal cells

All animal experiments were approved by the Bar-Ilan University Ethics Committee for Animal Research and were conducted in accordance with the Association for Research in Vision and Ophthalmology Statement for the Use of Animals in Ophthalmic and Vision Research. The retinas of Sprague Dawley P1 rats were isolated and dissociated using a papain dissociation kit (# LK003150, Worthington) according to the kit manual. Retinal cells were then incubated in a medium containing DMEM f12 (Gibco, United States) and neurobasal (Gibco) 1:1, glutamine 1% (Sigma-Aldrich), pen-strep 1%) Biological Industries, Beit-Haemek, Israel), non-essential amino acid 1%, and horse serum 2% (Biological Industries). B27, N2, and EGF (10 ng/μL, Peprotech, Israel), NGF (10 ng/μL, Peprotech), NT3 (10 ng/μL, Peprotech), BDNF (10 ng/μL, Peprotech), and FGF (10 ng/μL, Peprotech) were added to the medium. The medium was replaced after 24 h with fresh medium containing ROCK inhibitor (5 µL/1 mL, #1254/10, Biotest, Dreieich, Germany) and antimetabolite cytosine β-D-arabinofuranoside hydrochloride (Ara-c, 0.5 µL/1 mL, #C6645, Merck, United States), aiming to decrease the number of glia cells. Cells were incubated at 37°C with 5% CO_2_.

#### 2.4.2 HEK293-GFP

HEK293 cells were transfected with polyethyleneimine (PEI, Sigma Aldrich, 408,727) reagent dissolved in DDW (10 mg/1 mL) cells that were seeded on a 24-well plate. The transfection solution contained 0.5 µg DNA, 10 µL PEI, 70 µL DMEM per well, with the transfection plasmids’ CD-splice variant_pcDNA3.1 (+) IRES GFP_corrected and SCN1B-SCN2B_pcDNA3.1 (+) IRES (GenScript, NJ, United States) (Ben-Shalom et al., 2017). Human HEK293-GFP cell medium contains MEM-eagle (Biological Industries), 1% PSA (Biological Industries), 1% glutamine (Sigma-Aldrich), and 10% fatal bovine serum (Danyel biotech, Rehovot, Israel). Cells were incubated at 37°C with 5% CO_2_.

### 2.5 Cell adhesion assay

#### 2.5.1 Biomolecule’s effect on cell density

Aiming to assess the optimal biomolecule and concentration, the five types of biomolecules were diluted into four concentrations (1/3, 1/6, 1/12, and 1/24 mg/mL) in aqueous solutions DDW: Acetonitrile (1:1). The gold surfaces were coated as mentioned above in two micro-wells for each concentration (in duplicate). HEK-293-GFP and rat-dissociated retinal cells were seeded at an initial concentration of 0.8 M cells/mm^2^, as previously reported (Kleinfeld et al., 1988), and incubated for 24 h, similar to (Ritz et al., 2017). Then, the cells were gently rinsed with PBS to remove the unattached cells and fixed with 4% paraformaldehyde (#BN15711, Bar Naor, Israel) for 15 min at room temperature. Nuclear staining was performed with Hoechst (#14533, Sigma-Aldrich). Stained samples were rinsed (PBS) and mounted on slides in 90% glycerol (#G9012, Sigma-Aldrich)/10% PBS/1% n-propyl-gallate (#P3130, Sigma-Aldrich) and sealed with nail polish. The samples were imaged using a Leica LMD7 Microscope (Leica-microsystems, Germany) at a magnification of ×20. The navigation and stitching applications were used to extend the field of view by a factor of 9; three regions of interest (ROI) were imaged from the center of each micro-well, with two duplicates for each concentration, overall, with three repetitions. Cell density was calculated by ImageJ using a Gaussian filter (3-pixel mask) and binarization by the “Li dark” method (19–255). White (255) pixels were counted, after they referred to the fluorescence of the stained nucleus (no features were removed or added digitally). The average cell density was calculated from N = 18 fields for each concentration. To reduce the variability caused by various factors, for every repetition the cell density for each biomolecule was normalized to the cell density in the control condition (coating with DDW). An average of all repetitions and the standard error deviations (STDs) were calculated. Statistical significance was determined using either the paired two-tailed t-test or multi-variate two-way ANOVA analysis provided by the MATLAB statistics toolbox.

#### 2.5.2 Biomolecule’s effect on cell spreading

The cell area was measured as a function of the coated biomolecule. To this end, gold surfaces were coated by aqueous solutions (1/6 mg/mL, DDW: Acetonitrile, 1:1) of the biomolecule and the control as mentioned above with two micro-wells for each molecule (duplicates) and three repetitions. HEK-293-GFP and rat-dissociated retinal cells were seeded at an initial concentration of 0.15 M cells/mm^2^ to allow single-cell spreading and incubated for 72 h. For the rat-dissociated retinal cells, 10 µM Ara-c and 50 µM ROCK inhibitors were added after 24h, aiming to restrict the glial cell growth (Kleinfeld et al., 1988). Then they were gently rinsed with PBS to remove unattached cells; the rat-dissociated retinal cells were stained for cytoplasmic staining using ViaFluor^®^ 488 (#BTM-30086, 1:4,000, Biotium, Fremont, CA, United States) following the supplier protocol, fixed with 4% paraformaldehyde (#BN15711, Bar Naor, Israel) for 15 min at RT, and nuclear staining was performed by Hoechst (#14533, 1:1,000, Sigma- Aldrich). Stained samples were rinsed (PBS) and mounted on slides in 90% glycerol (#G9012, Sigma-Aldrich)/10% PBS/1% n-propyl-gallate (#P3130, Sigma-Aldrich) and sealed with nail polish. Untouched single cells were imaged for each sample using a Leica LMD7 Microscope (Leica-microsystems, Germany) at a magnification of ×63. The cell surface area of n = 8 cells was calculated based on the cytoplasmic images (green channel, 532 nm) by ImageJ, as was previously proposed (Truskey and Proulx, 1990). Briefly, the images were blurred by a Gaussian filter (3-pixel mask) and binarized by a “Li dark” filter (no features were removed or added digitally).

The average cell surface area was measured by counting the fluorescence pixels and dividing them by the number of nuclei in the frame; this was further normalized to the average surface area of the “DDW coated” images. Statistical analysis was determined using both multi-variate one-way ANOVA analysis and a two-tailed t-test, provided by the MATLAB statistics toolbox.

#### 2.5.3 Biomolecule effect on the focal adhesion spots

The effect of the various biomolecules on the focal adhesion complex formation was estimated by immunocytochemistry. To this end, gold surfaces were coated by aqueous solutions with an intermediate concentration (1/6 mg/mL, DDW:AN, 1:1) of the various biomolecules and the control, as mentioned above. This intermediate concentration was chosen since statistical analysis revealed no significant effect of concentration on cell density. HEK-293-GFP and rat-dissociated-retinal cells were seeded at an initial concentration of 0.8 K cells/mm^2^ to allow single cell spreading and incubated for 72 h (for rat-dissociated retinal cells 10 µM Ara-c and 50 µM ROCK inhibitor were added after 24 h). Following incubation, cells were immuno-stained for cytoskeletal markers as follows: Surfaces were rinsed gently with PBS to remove unattached cells; then the cells were fixed with 4% paraformaldehyde (Bar Naor, BN15711) for 15 min at room temperature. Next, the cells were rinsed in PBS with 0.5% Triton-X100 (Amersham, 22686) and 1% Tween (Amersham, 20605) (PBST). Blocking was performed for 30 min in a blocker solution containing 1% bovine serum albumin (MP Biomedicals, 160069). Cells were incubated overnight at 4°C with primary antibodies for anti-Vinculin antibody (Cavalcanti-Adam et al., 2007) (mouse, #V9131, Sigma-Aldrich, 1:50); the retinal cells were further incubated with anti-CRX antibody (rabbit, #NBP2-15964, Biotest, 1:100). The next day the cells were rinsed with PBST, and secondary antibodies coupled to Alexa Fluor 594 anti-mouse (Jackson ImmunoResearch, #711-585-152) and Alexa Fluor 488 anti-rabbit (Jackson ImmunoResearch, #711-545-152) were applied for 30 min at RT and washed with PBS. F-actin staining was performed with #P5282, Sigma-Aldrich, 1:500 and nuclear staining was performed with Hoechst (Sigma-Aldrich, 100 MG-14533). Stained samples were rinsed in PBS, mounted on slides in 90% glycerol (Sigma-Aldrich, G9012)/10% PBS/1% n-propyl-gallate (Sigma-Aldrich, P3130), and sealed with nail polish. Cells were imaged using a Leica-Stellaris-5 microscope (Leica-microsystems, Germany) at ×100 oil immersion objective. Aiming to estimate the focal adhesion (FA) spots, the Vinculin channel was binarized using ImageJ and the bright spots for each cell were counted. A DDW-normalized average amount of FA for each molecule was calculated, and the statistical significance was determined using both multi-variate one-way ANOVA analysis and a two-tailed t-test, provided by the MATLAB statistics toolbox.

#### 2.5.4 Evaluation of the gene expression

Quantitative PCR analysis was used to evaluate the regulation pathways of focal adhesion (Ritz et al., 2017; Schick et al., 2020). Briefly, HEK293-GFP and rat-dissociated retinal cells were seeded on different biomolecule-coated gold surfaces (1/6 mg/mL), (60 M cells in total for each repetition). RNA was extracted after 72 h of incubation using the Gene Elute Mammalian Total RNA Miniprep Kit (#RTN70, Sigma-Aldrich) according to the manufacturer’s instructions. RT-PCR was performed on RNA extracted from the cells, which was synthesized to cDNA using M-MLV reverse transcriptase (#M1701, Promega, Madison, WI, United States). Quantitative PCR analysis was then performed using PerfeCTa SYBR Green FastMix (#95074-250, Quantabio, Beverly, MA, United States).

The primers were designed according to the gene sequences database of the National Library of Medicine website (https://www.ncbi.nlm.nih.gov/gene/), and the specificity of each primer was assessed using the BLAST software and UCSC In-Silico PCR (https://genome.ucsc.edu/cgi-bin/hgPcr). Primers were manufactured by Sigma Aldrich, checked via gel electrophoresis, and melting curves at 60°C; the efficiency and specificity were assessed using linear calibration curves at various concentrations. The corresponding primers of the genes of interest are listed in Table Supp-T1 in the Supplementary Material.

We focused on investigating the gene expression of several adhesion integrin subunits, namely, integrin α_IIb_ (ITGA2B), integrin α_V_ (ITGAV), integrin α_5_ (ITGA5), integrin β_1_ (ITGB1), integrin β_3_ (ITGB3), and the focal adhesion-associated proteins Vinculin and protein tyrosine kinase 2 (PTK-2). The expression level of the gene of interest was compared to glyceraldehyde 3-phosphate dehydrogenase (GAPDH), normalized to the expression level of cells seeded on the control group (DDW coating), and was then calculated by the Pfaffl method, where the primer reaction efficiencies were extracted from the primer’s linear calibration curve. Multivariable one-way ANOVA test, N-way ANOVA, and a t-test were performed.

### 2.6 Data analysis

Data are expressed as means ± Standard Deviation (SD) and were calculated from the results of at least three independent experiments. Two-way ANOVA and one-way ANOVA were performed for the density experiment, one-way ANOVA for the cell spreading and the FA spot number, N-way ANOVA for the gene expression experiment, and Student’s t-test for all the experiments compared to DDW and to the Optimal biomolecule as described in each section. A value of *p* < 0.05 was considered as statistically significant.

## 3 Results

### 3.1 Surface modification

**FIGURE 2:**
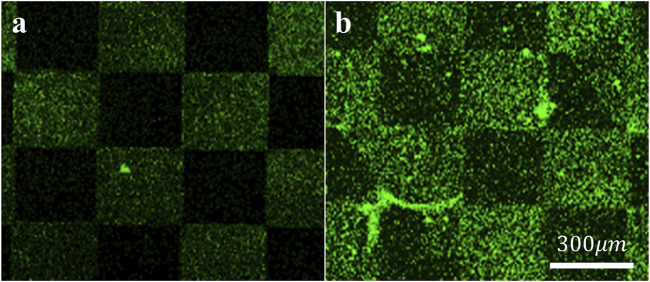
Assembly of the biomolecules to gold surfaces. STED Fluorescent images of **(A)** untreated and **(B)** fluorescent RGD-NBD-coated glass-gold patterned surfaces revealed RGD assembly mainly in the gold areas. Scale bar 300 µm.

**FIGURE 3:**
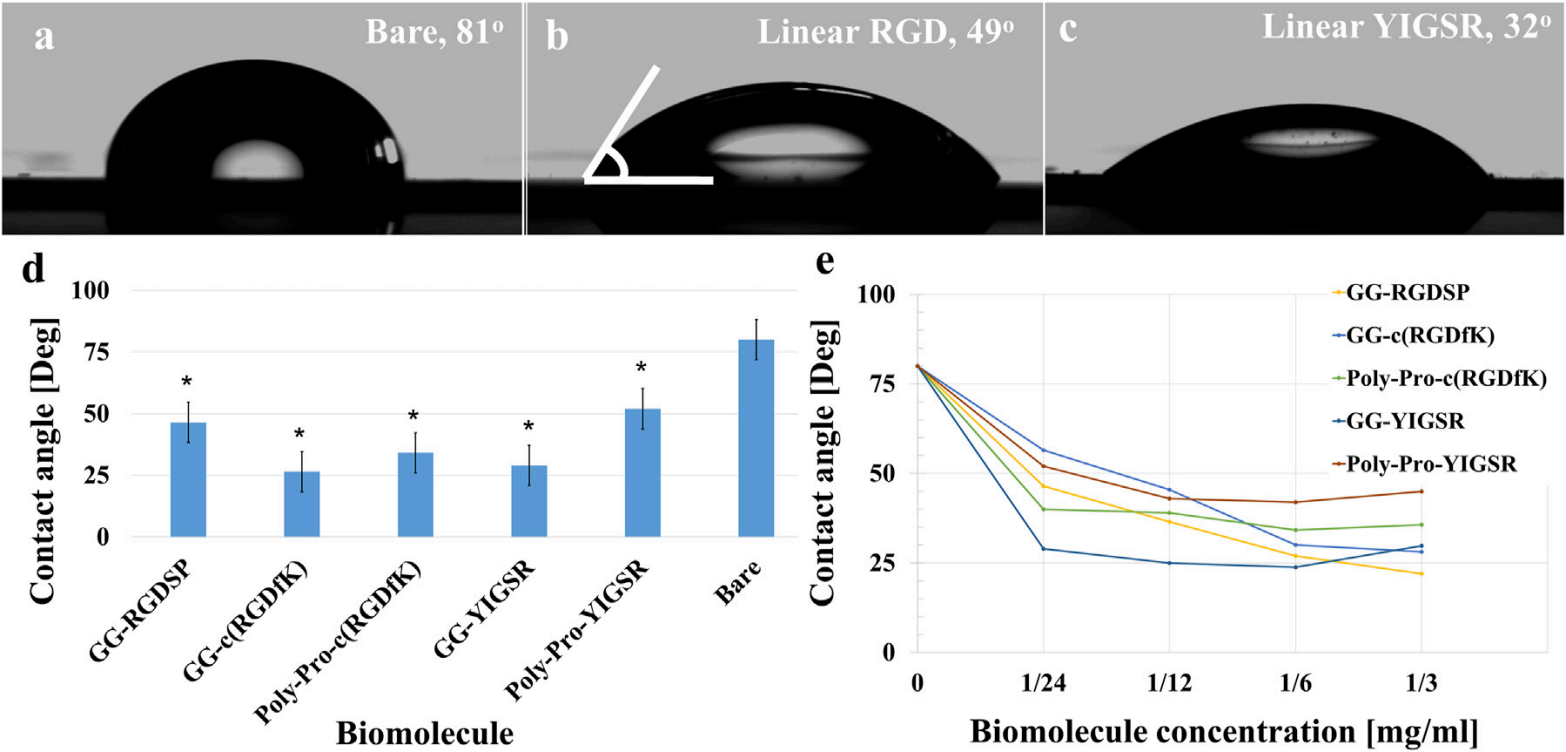
Contact angle measurements presenting gold surface modifications. **(A–C)** Contact angle images of untreated **(A)**, short linear RGD coated **(B)**, and short linear YIGSR **(C)** coated gold surfaces. **(D)** The water contact angle of gold surfaces coated with the various molecules (N = 3). *p < 0.05 compared with bare gold. **(E)** The effect of the biomolecule concentration on the contact angle (N = 3), *p* < 0.05.

To assess the specific assembly of the biomolecules to the gold surface, we used a fluorophore paired RGD molecule (NBD-RGD as a representative molecule) with a Cys anchor to coat a checkerboard-patterned gold-glass surface. Figure 2 shows a clear checkerboard green-fluorescent pattern with higher fluorescence in the gold compared with bare glass, suggesting the specific binding of the biomolecule through the thiol group to the gold; these results are in agreement with previous reports (Bendali et al., 2014). X-ray photoelectron spectroscopy (XPS) of the Cys-RGD-coated gold surface (Supplementary Figure S1) revealed energy peaks at 162.5 eV and 397 eV, which are known to fit S_2p_ (sulfur-gold) and N_1s_ (metal nitrides) energy, respectively, indicating the coating of the gold surface by the peptide biomolecule (Moulder et al., 1992). Contact angle measurements between the gold surface and a water droplet (Figures 3A–C) were used to assess the surface energy and wettability relating to the hydrophilicity of the surface. Since the biomolecules are hydrophilic peptides containing amide and carboxylic groups on their chain side, they elicited a decrease in the contact angle compared with the untreated (bare) gold surface, as is shown in Figures 3A–C. All biomolecules used in the current project showed a similar decrease in contact angle compared with the bare gold surface (*p* < 0.05); the lowest contact angle was measured for linear short RGD at 22.2° (Figure 3D). The contact angle decreased with increasing biomolecule concentration, reaching a plateau at 1/12 mg/mL (Figure 3E). The results of the gold and gold thiol-RGD coatings are in the order of the result previously published (Yoon and Mofrad, 2011).

### 3.2 Retinal cells are attracted by YIGSR biomolecules

To evaluate the optimal biomolecule and its concentration for promoting cell adhesion in general, and retinal cells in particular, gold surfaces were coated with the studied biomolecules at concentrations of (Pallarola et al., 2017; Verrier et al., 2002) (1/24 mg/mL, 1/12 mg/mL, 1/6 mg/mL, and 1/3 mg/mL); gold surfaces soaked in DDW served as controls. Both cell types were seeded (0.8 K cells/cm^2^) and incubated for 24 h onto the pre-coated surfaces. The normalized cell density was evaluated by a self-coded ImageJ image-processing tool (as described in the Methods section) by dividing the number of nuclei counted in each figure by its surface area and by normalizing the average cell density of each biomolecule by the cell density of DDW.

**FIGURE 4:**
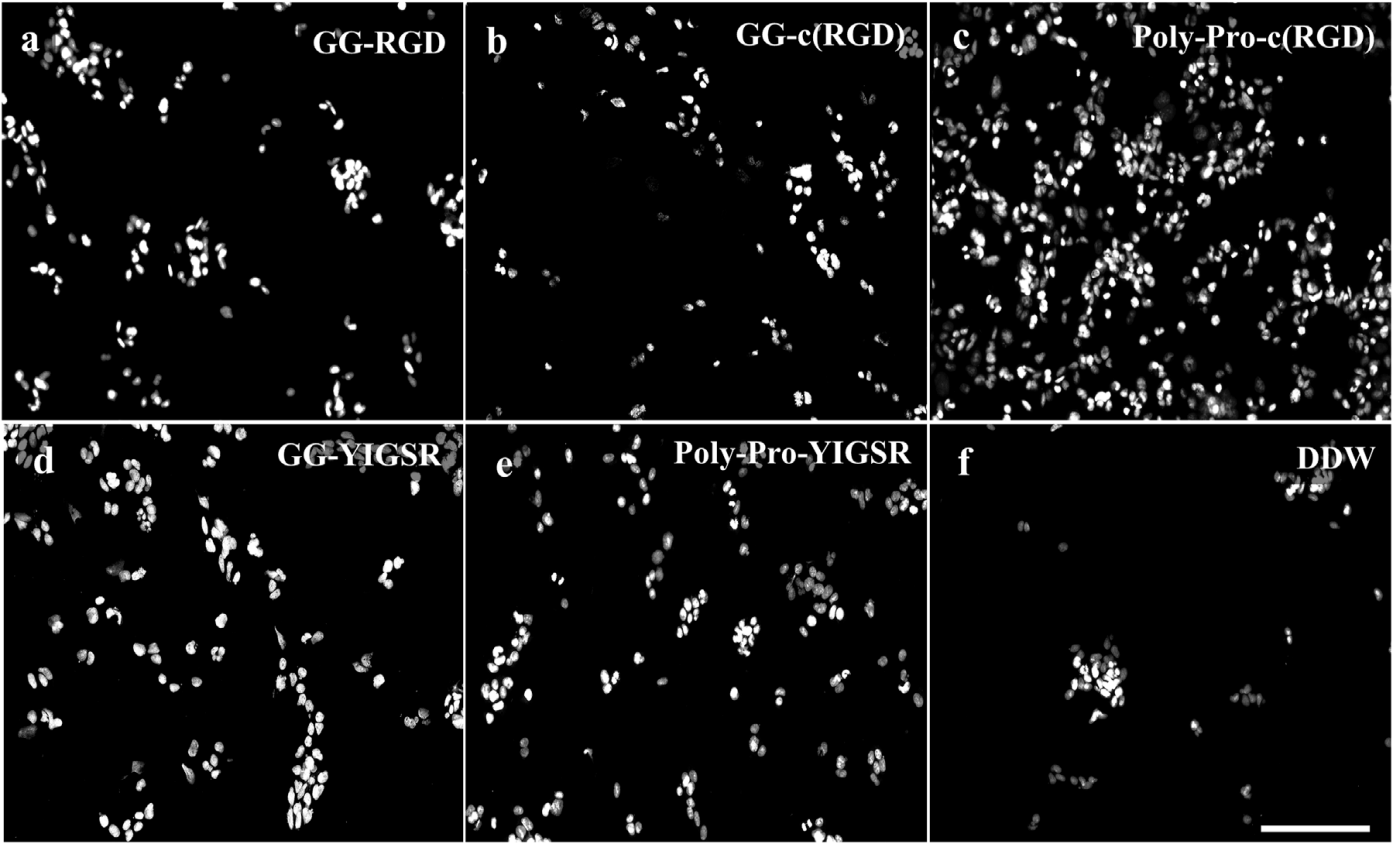
Effect of various biomolecule coatings on the HEK293 cells. **(A)** Short linear RGD, **(B)** short cyclo-RGD, **(C)** long cyclo-RGD, **(D)** short YIGSR, **(E)** long YIGSR, and **(F)** DDW. Cells were seeded on the coated gold surface with a biomolecule concentration of 1/3 mg/mL and incubated for 24 h, followed by fixation and Hoechst staining. Scale bar 200 µm. (N = 18).

**FIGURE 5:**
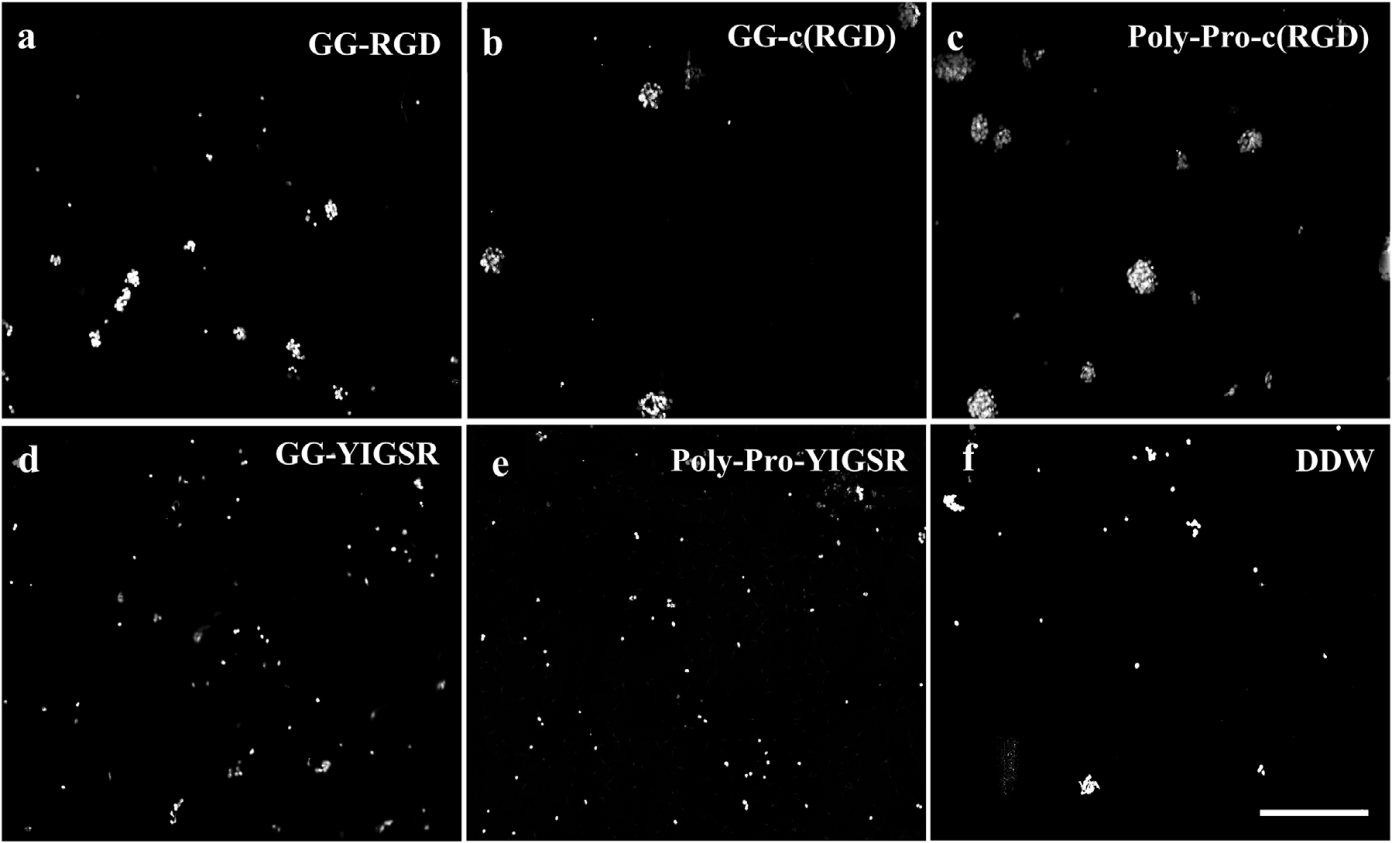
Effect of various biomolecule coatings on the retinal cells. **(A)** Short linear RGD, **(B)** short cyclo-RGD, **(C)** long cyclo-RGD, **(D)** short YIGSR, **(E)** long YIGSR, and **(F)** DDW only. Cells were seeded on the coated gold surface with a biomolecule concentration of 1/3 mg/mL and incubated for 24 h, followed by fixation and Hoechst staining. Scale bar 200 µm.

Figures 4, 5 present representative images of HEK293 and retinal cells, respectively, cultured on gold surfaces coated with the studied biomolecules at a concentration of 1/3 [mg/mL], suggesting that all investigated biomolecules improved the adhesion of both cell types compared to DDW; specifically, HEK293 cells were mainly attracted to the RGD-type biomolecules, whereas the retinal cells were mainly affected by the YIGSR-type molecules.

**FIGURE 6:**
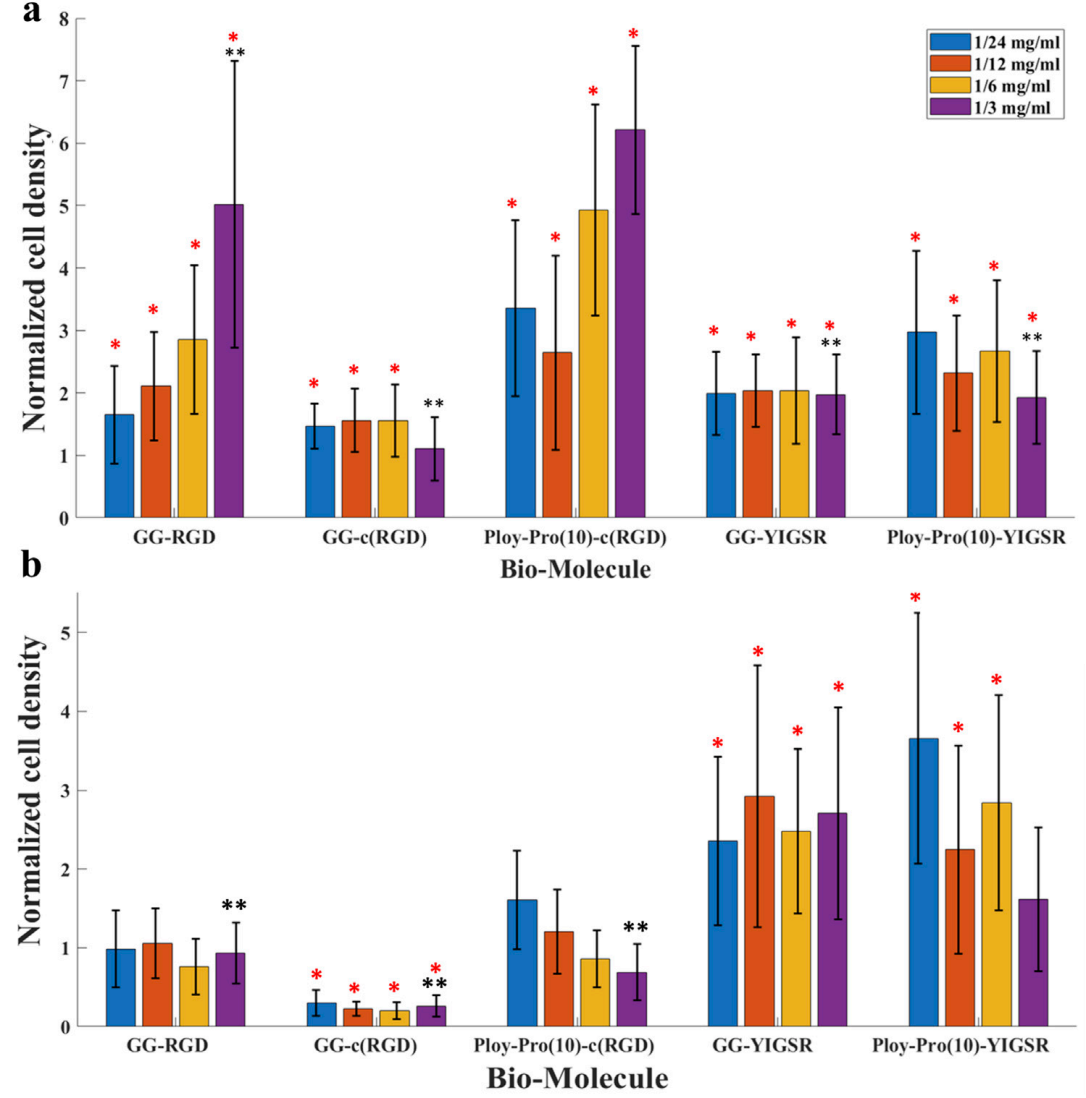
The effect of biomolecules on cell density. **(A)** HEK 293 cells and **(B)** rat retinal cells. Cells were seeded on gold surfaces coated with various biomolecules at several concentrations. Cell density was evaluated using a Leica LMD7 microscope and a binarization ImageJ algorithm. For each biomolecule, the cell density was normalized to a cell density of DDW. *Red star denotes significance compared to control, *p*<<0.05. **Black star denotes significance compared to a long cyclo-RGD [Poly-Pro-c (RGD)]-coated surface, *p*<<0.05.

Quantification of the normalized average (N = 18, normalized to DDW) cell density of HEK293 and retinal cells cultured on the various surfaces is shown in Figures 6A, B, respectively. For the HEK293 cells (Figure 6A), all biomolecules elicited a higher cell density compared with DDW (one way ANOVA *p* < 0.001 for all biomolecules). The highest cell density was observed for the long-spacer cRGD of 6.2 ± 1.3 (*p* < 0.005 for comparison with all other molecules), followed by the short spacer non-cyclic RGD (GG-RGD, (5.02 ± 2.3). For these two biomolecules, cell density significantly increased with increasing biomolecule concentration (both with P for trend = 0.001).

In contrast with the HEK293 cells, the retinal cells (Figure 6B) were mainly attracted to the YIGSR ligand. The highest cell density was found for the long spacer YIGSR (Poly-Pro-YIGSR) at the lowest concentration (3.6 ± 1.6), *p* < 0.001 compared with DDW). However, an n-way ANOVA investigation suggested that there was no significant difference between the different concentrations (*p* > 0.19). A slightly lower cell density was observed for the short spacer YIGSR (2.6 ± 1.2, *p* = 0.001 compared with DDW). The T-test comparison between all the biomolecule permutations for both HEK293 and retinal cells with a concentration of 1/3 [mg/mL] is presented in Supp T2-3. These results highlight the superiority of the long spacer cyclo-RGD molecule for HEK293 cells and the YIGSR molecule for retinal cells regarding cell density and adhesion.

### 3.3 YIGSR biomolecules elicited retinal cell spreading

**FIGURE 7:**
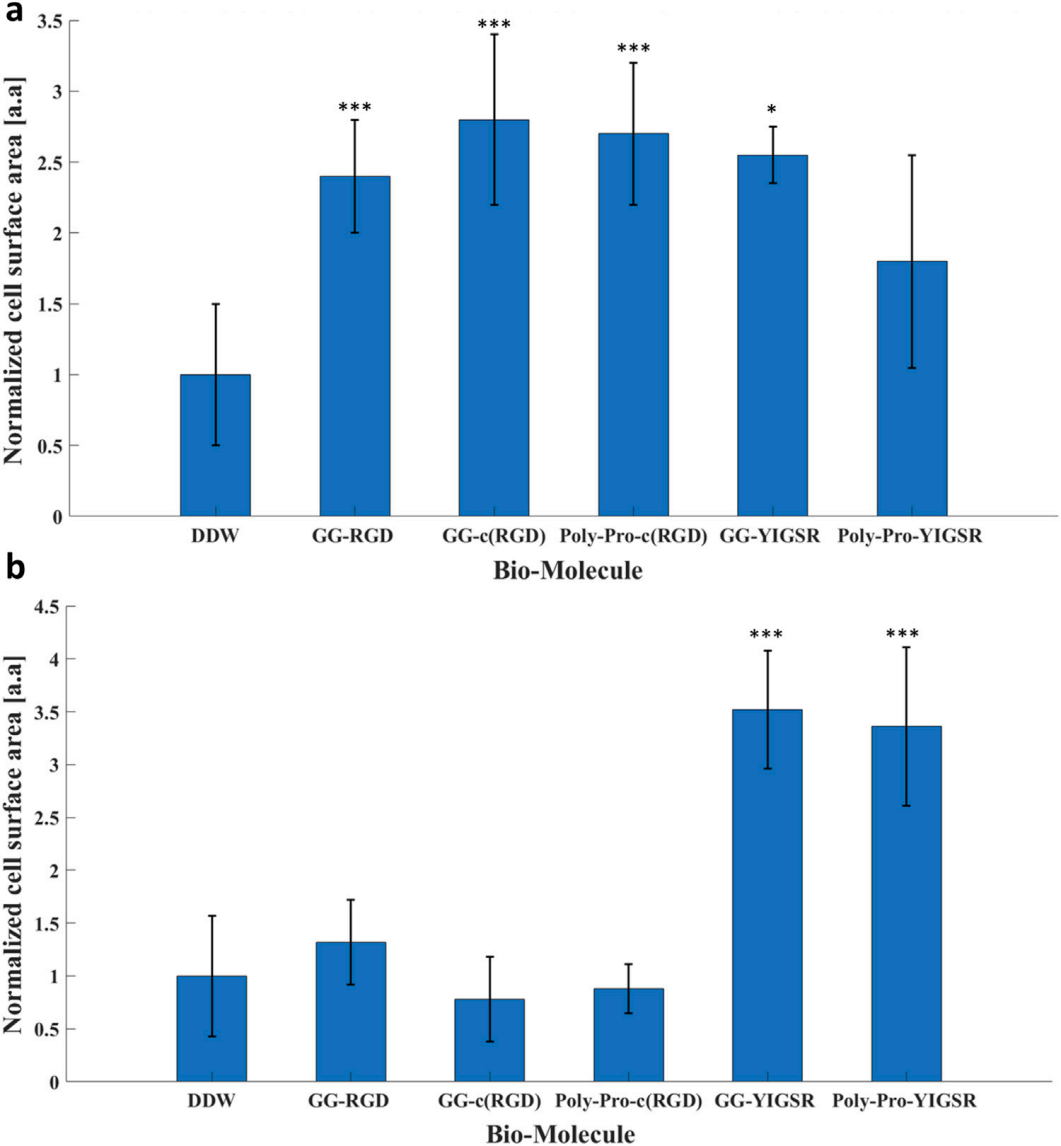
The effect of the coating molecules on the cell surface area. **(A)** HEK293 cells and **(B)** rat retinal cells. The cells were incubated for 72 h after being seeded on various biomolecule-coated gold surfaces (1/6 mg/mL) followed by fixation and staining. The average surface area from each biomolecule-coated surface was normalized to that of DDW. *p < 0.05, ***p < 0.001, compared to untreated (bare) gold.

As an additional measure of the effect of the biomolecules on cell-surface interaction, we investigated the effect of the biomolecule on the cell surface area (Massia and Hubbell, 1991). To this end, both types of cells were seeded on gold surfaces coated with various biomolecules and incubated for 72 h, after which the rat retinal cells were stained for Viafluor cytoplasmic indicator; the HEK293 cells constitutively expressed GFP. The average cell surface was calculated by ImageJ and normalized based on the cell surface measurements obtained for DDW (Figure 7). Quantitative analysis showed that for the HEK293 cells (Figure 7A), both the RGD and YIGSR molecules led to a significant increase in the cell surface area. The largest cell surface area was observed with the cyclic molecules (2.7 ± 0.7 and 2.6 ± 0.8 for GG-c (RGD) and poly-pro-c (RGD), respectively, *p* < 0.001 compared with DDW). Similarly, the cells seeded on gold coated with GG-YIGSR molecules exhibited an increased surface area compared with those treated with DDW (2.5 ± 0.1, *p* < 0.05 compared with DDW).

In contrast with the HEK293 cells, for the retinal cells, only the YIGSR biomolecules (Figure 7B) elicited a significant increase in the normalized surface area; an increase of 3.5 ± 0.6, and 3.4 ± 0.7-fold was found for the short and long spacer molecules, respectively (*p* >>0.001 compared with DDW). Representative images of the cell spreading are presented in the Supplementary Figures S2, S3. In addition, T-test comparison between all biomolecule permutations is presented in Supplementary Tables S4, S5. These results are in alignment with the differential effect of YIGSR and RGD on the cell density observed for HEK293 and the retinal cells, further stressing the importance of a cell-specific coating design for implantable devices and electrodes.

### 3.4 Focal adhesion spots

**FIGURE 8:**
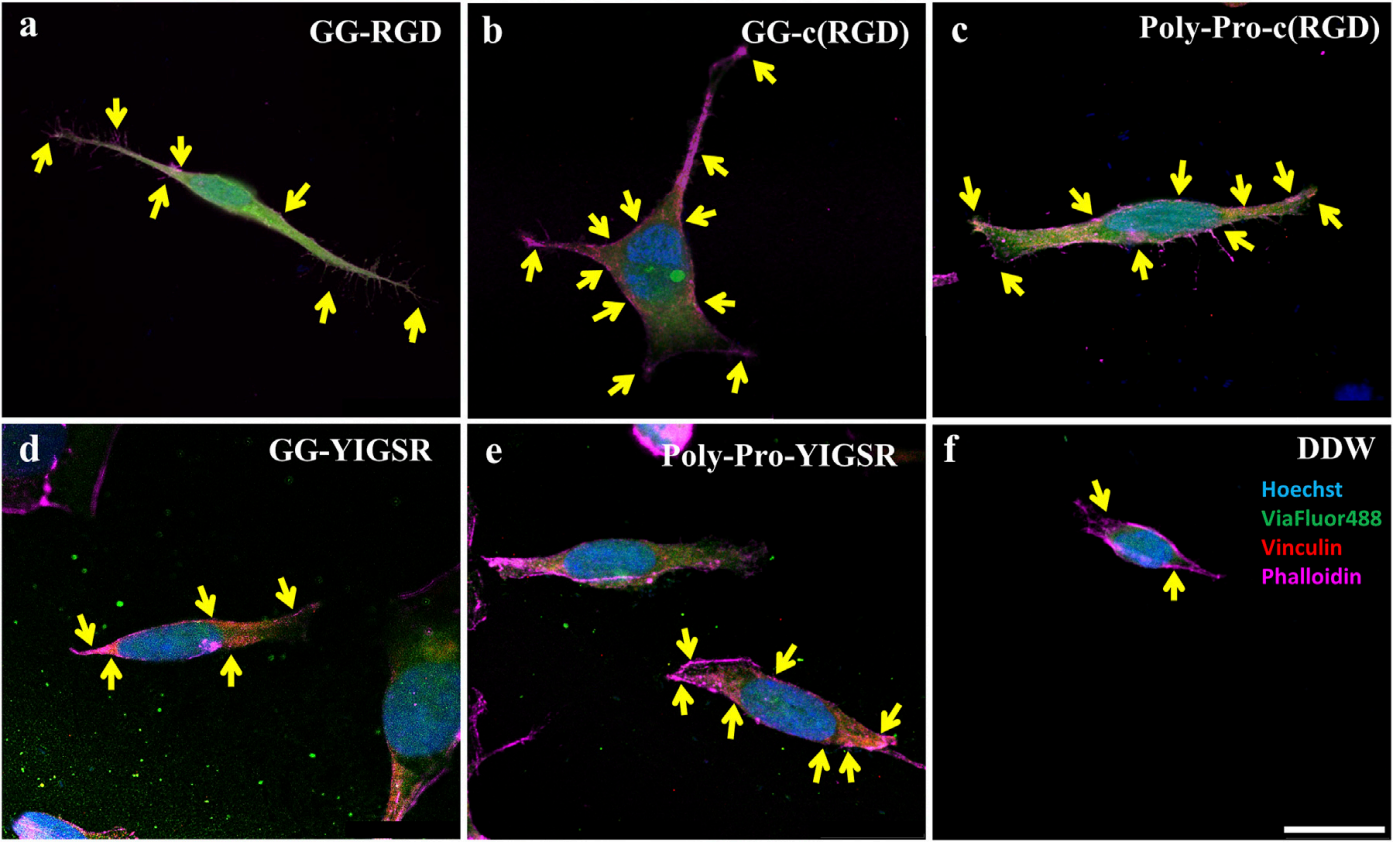
Confocal images of HEK293 cells presenting the effect of coating biomolecules on focal adhesion spots. Cells were incubated for 72 h after being seeded on various biomolecule-coated gold surfaces (1/6 mg/mL) followed by fixation and staining. **(A)** Short linear RGD, **(B)** short cyclo-RGD, **(C)** long cyclo-RGD, **(D)** short YIGSR, **(E)** long YIGSR, and **(F)** DDW only. Biomolecule concentration of 1/12 mg/mL. Yellow arrows denote spots of focal adhesions. Hoechst (Blue), genetically encoded GFP (green), Vinculin (FA, red), f-actin (magenta). Scale bar 20 µm.

**FIGURE 9:**
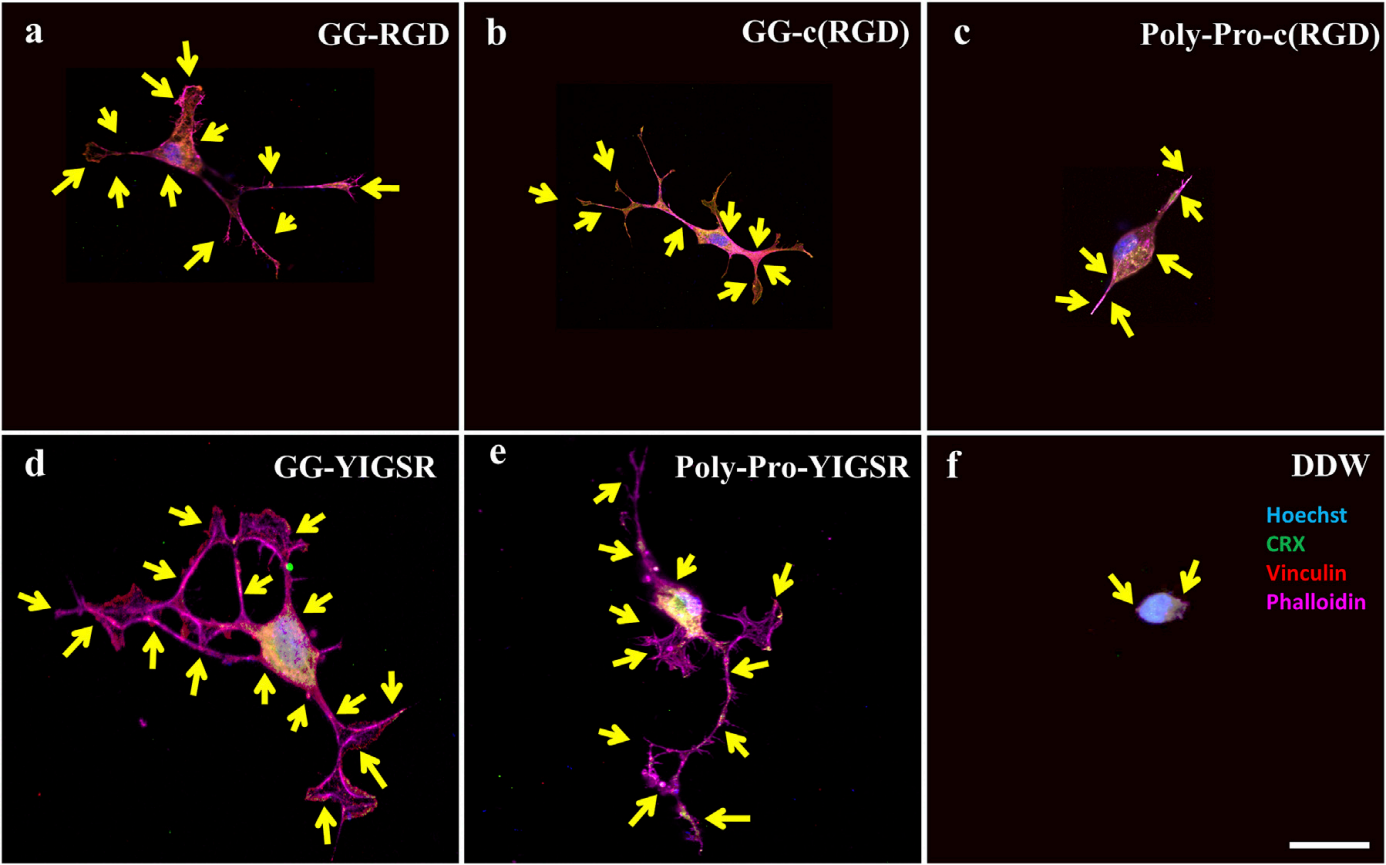
Confocal images of rat-dissociated retinal cells presenting the effect of coating biomolecules on the focal adhesion spots. Cells were incubated for 72 h after being seeded on various biomolecule-coated gold surfaces (1/6 mg/mL), followed by fixation and staining. **(A)** Short linear RGD, **(B)** short cyclo-RGD, **(C)** long cyclo-RGD, **(D)** short YIGSR, **(E)** long YIGSR, and **(F)** DDW only. Biomolecule concentration of 1/12 mg/mL. Yellow arrows denote spots of focal adhesions. Hoechst (blue), ViaFluor488 (green), Vinculin (FA, red), f-actin (magenta). Scale bar 25 µm.

We also studied the effect of the studied biomolecule coating on the focal adhesion (FA) mechanism. To this end, both cell types were seeded on pre-coated gold surfaces with various biomolecules (1/6 mg/mL), fixed and stained for Vinculin (the main protein in the FA complex), the cytoskeleton F-actin filament (also integrated into the FA complex), and the nuclei. FA spots (Vinculin clusters) were counted manually (Figures 8, 9, arrows).

**FIGURE 10:**
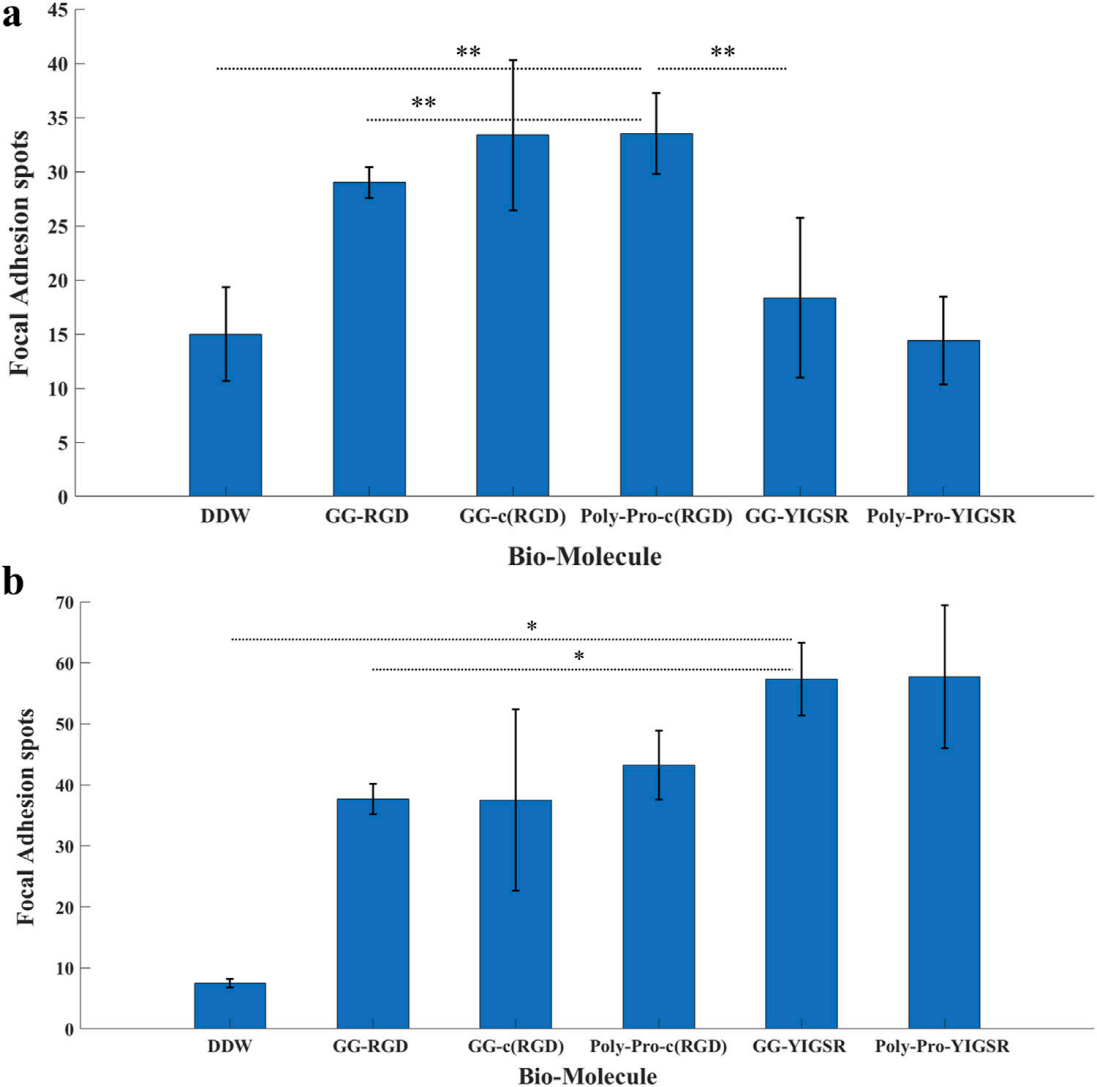
The effect of coating molecules on the focal adhesion spots. **(A)** HEK293 cells and **(B)** retinal cells. The cells were incubated for 72 h after being seeded on various biomolecule-coated gold surfaces (1/6 mg/mL) followed by fixation and staining. The number of bright Vinculin spots was counted manually. *p < 0.03, **p < 0.001.

Figures 8, 9 depict characteristic immunocytochemistry confocal images for both cell types; yellow arrows indicate representative FA spots. Figure 8 suggests that the RGD molecules affected the HEK293 surface attachment, showing developed filopodia and cell spreading (Figures 8B, C). In contrast, YIGSR affected the rat-dissociated-retinal cells (Figures 9D, E) more than the RGD-type molecules (Einer and Elner, 1996). Quantitative analysis of the average cellular number of FA spots is presented in Figure 10, for HEK293 (Figure 10A) and retinal cells (Figure 10A). In line with the results obtained for HEK293 regarding the cell density and cellular area (Figures 6, 7), the cyclic RGD molecules elicited significantly more FA spots compared with the YIGSR molecules (*p*<<0.01 for both cyclic RGD vs. both YIGSR and DDW). The largest effect was with the cyclic molecules (33.3 ± 6.9, *p* < 0.01, and 33.5 ± 3.7 *p* < 0.01, compared with DDW for short (GG-cRGD) and long (Poly-Pro-cRGD) spacers, respectively); the short linear (GG-RGD) elicited 29 ± 1.4 focal adhesion spots (*p* < 0.01 compared with DDW). The average cellular number of FAs elicited by the YIGSR molecules was 18.3 ± 7 and 14.4 ± 4.0 spots for the short (GG-YIGSR) and long spacer (Poly-Pro-YIGSR) molecules, respectively, not significantly higher compared with DDW (*p* > 0.4).

In contrast with the HEK293 cells, for the retinal cells (Figure 10B), all biomolecules elicited a significant increase in the average cellular FA spots, compared with DDW; the largest effect was observed for short-spacer YIGSR (GG-YIGSR, 82.0 ± 15.7, *p* = 0.02 compared with DDW, *p* = 0.04 compared with GG-RGD). Detailed T-test comparison between all coated biomolecules for both HEK293 and retinal cells is presented in the Supplementary Tables S6, S7. These results further support YIGSR as the biomolecule of choice for attracting retinal cells.

### 3.5 Gene expression

**FIGURE 11:**
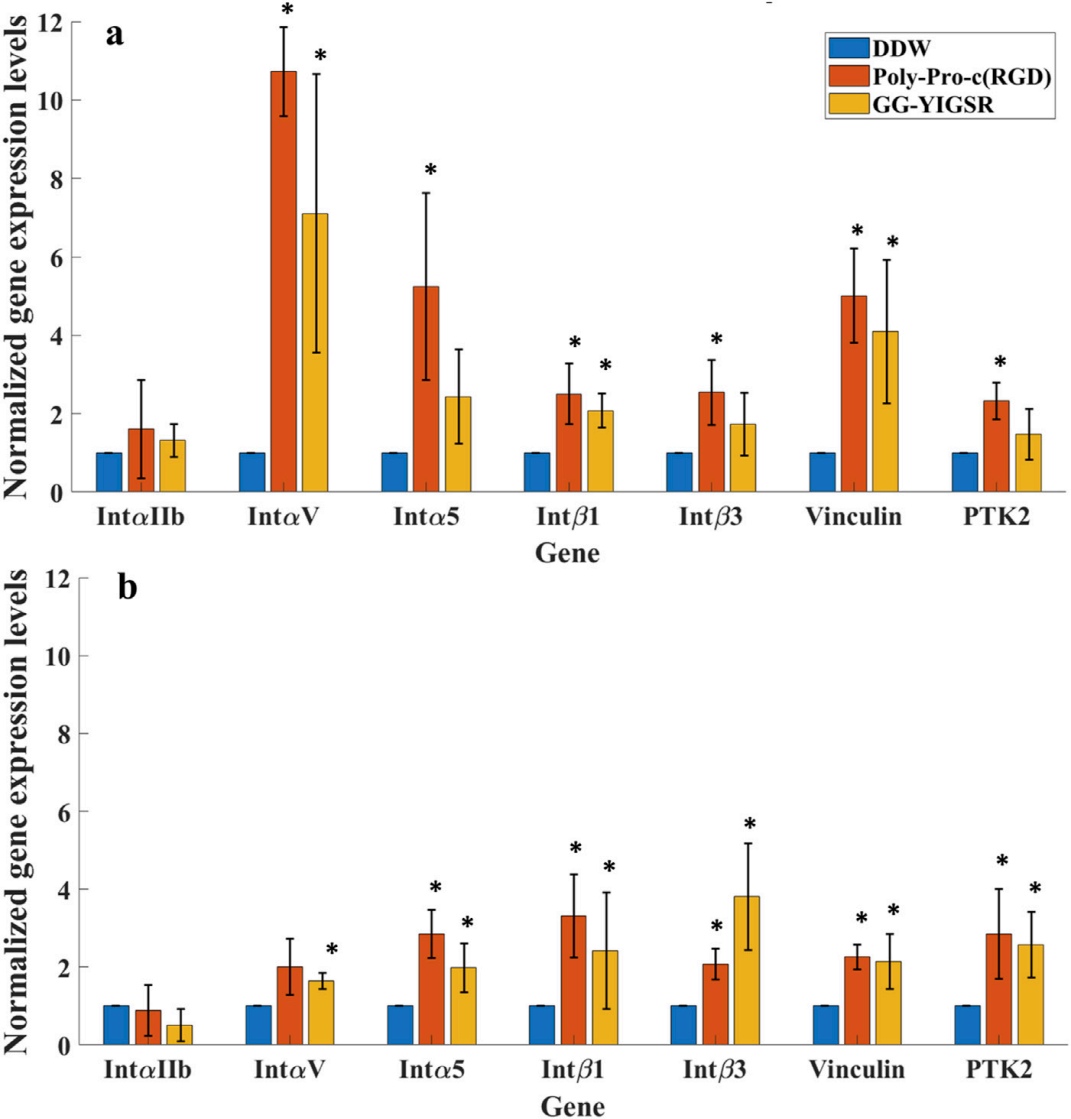
qPCR analysis of the Effect of surface coating on the Relative normalized gene expression levels of adhesion integrins and focal adhesion proteins. **(A)** HEK293 cells. **(B)** Rat-dissociated retinal cells. Cells were incubated for 72 h after being seeded on various biomolecule-coated gold surfaces (1/6 mg/mL) before RNA extraction. Expression levels were normalized to the expression level of the GAPDH gene, used as a reference gene. *p < 0.05 compared to DDW.

To better understand the molecular mechanism by which the coating biomolecules regulate cell adhesion via the adhesion integrins and focal adhesions, we quantified the expression of genes involved in cell adhesion (Perdih and Sollner Dolenc, 2010; Rao and Winter, 2009). We performed real-time qPCR of genes associated with various subunits of adhesion integrins (Intα_IIb_, Intα_V_, Intα_5_, Intβ_1_, and Intβ_3_) and focal adhesion proteins (Vinculin and PTK-2) for cells seeded on gold surfaces that were coated with the biomolecules (1/6 mg/mL). In these experiments, we focused on the molecules that were found to be optimal in the previously described experiments, namely, a long spacer with cyclic-RGD (Poly-Proline-c (RGD) and short spacer YIGSR (GG-YIGSR). Results were normalized to cells seeded on the untreated gold surface after being normalized to GAPHD expression. The relative normalized gene expression levels for both cell types are presented in Figure 11.

Both biomolecules amplified most of the examined adhesion genes for both cells. For the HEK293 cells (Figure 11A), most of the adhesion integrins and the focal adhesion genes were affected by the two biomolecules. In general, the effect of RGD was higher than with YIGSR (N-Way ANOVA, *p* < 0.005). The largest effect of RGD coating on the HEK293 cells’ gene expression was observed for Intα_V_, Intα_5,_ and Vinculin (10.7 ± 1.4, 5.2 ± 2.3 and 5.0 ± 1.2-fold, t-test *p* = 0.001, 0.03, and 0.004, respectively). For the retinal cells, the effect of the various biomolecules on the expression levels of the adhesion integrins and the focal adhesion proteins (Figure 11B) was a mild increase in the various integrins except for Intα_IIb_.

## 4 Discussion

Various types of integrins are widely expressed in the retina and play a crucial role in mediating retinal cell attachment to extracellular matrix (ECM) proteins during retinal development and axon growth (Einer and Elner, 1996; Brem et al., 1994). Some research suggests that integrins are involved in ocular diseases, offering a potential therapeutic target (Aktas et al., 2024); others propose leveraging their mechanism to enhance cell adhesion to the scaffold in retinal cell patches or other applications through biomaterial scaffolds that incorporate their ligands (Zhao and Du, 2022; Treharne et al., 2011). Although neural adhesion by integrins or other cell adhesion molecules has been investigated before (Unsworth et al., 2011), only a few studies have been conducted regarding their incorporation with retinal cells [e.g., chicken retinal ganglion cells (Huber et al., 1998)]. The current work investigated the ability to promote retinal cell adhesion by the self-assembly monolayer (SAM) of bio-peptidomimetics, which imitates the ECM adhesion motifs and promotes FA formation; this could play an important role in improving future neuron-electrode interfaces in electronic retinal prostheses or for devising a scaffold integrated with retinal cells. To this end, gold surfaces, which were used for mimicking electrodes, were coated using various short biomolecules. We investigated the optimal molecule design regarding its ligand head sequence (e.g., RGD or YIGSR), its spatial conformation (linear or cyclic), and the spacer length (short and long). We hypothesized that cell adhesion occurs through focal adhesion formation, which is regulated via the integrin mechanism, specifically Intα_5_β_1_ and Intα_V_β_3_ (Perdih and Sollner Dolenc, 2010; Mas-Moruno et al., 2016; Hersel et al., 2003), and involves the overexpression of adhesion integrins and focal adhesion protein genes. The cell adhesion over the biomolecules was investigated by measuring the cell density, the cell area (spreading), the number of focal adhesion sites, and adhesion-related gene expression compared to untreated gold surfaces; all experiments were performed for rat-dissociated retinal cells and human HEK293.

Surface analysis by contact angle analysis revealed an increase in the surface’s hydrophilic nature after surface modification; the fluorescence images also demonstrated the selectivity of the molecules to gold surfaces, similar to previous reports (Yoon and Mofrad, 2011). Next, aiming to investigate the optimal biomolecule type and concentration, the cell density was evaluated after 24 h post-seeding. Our results showed that retinal cells were attracted to surfaces coated with the YIGSR molecules, whereas the HEK293 cells were mainly attracted to the RGD-type molecules (GG-RGD and poly-pro-cRGD). These results highlight the cell-specific nature of cell adhesion by the integrin mechanism. Previous studies have shown that YIGSR biomolecules specifically elicited neural cells’ (Rao and Winter, 2009; Jain and Roy, 2020) attachment to surfaces. Our results show for the first time the superiority of YIGSR molecules for attaching retina cells to surfaces.

Previous studies (Verrier et al., 2002; Porté-Durrieu et al., 2004) showed the superiority of cyclic-RGDfK on linear RGDfK in promoting cell adhesion. Similarly, our results showed the benefit of cyclic RGD on linear RGD on HEK293 cells’ focal adhesion; this effect was found for the cell density for the long spacer cRGD, similar to previous studies (Pallarola et al., 2017; Pallarola et al., 2014). However, this effect was not found for the retinal cells.

Furthermore, we explored the cell spreading area as another characteristic of cell adhesion. Our results are consistent with the density results where the two YIGSR-type molecules significantly increased the retinal cell surface area, whereas the RGD-type molecules did not. Interestingly, for the retinal cells, the spacer length of the YIGSR molecule does not seem to have any effect on the cell spreading. This result is contrary to previous studies reporting the superiority of a long spacer compared to a short spacer with YIGSR molecules on neural neurite outgrowth (not the surface area); this further demonstrates the cell-specific nature of the cell adhesion mechanism (Tong and Shoichet, 2001). In contrast with retinal cells, for HEK293, all molecules enhanced the cell spreading; the maximal effect was found for the RGD-type molecules, in alignment with previous reports (Cavalcanti-Adam et al., 2007; Cavalcanti-adam et al., 2006).

To verify our assumption regarding the recruitment of focal adhesion proteins to promote cell adhesion, we quantified the FA spots, as indicated by Vinculin clusters in confocal images 72 h post-seeding. Indeed, we found that the adhesion biomolecule-coated electrode surfaces stimulated [presumably via cellular integrins (Mitsios et al., 2010; Wang et al., 2021)] the recruitment of cytoplasmic proteins (e.g., Vinculin) (Mitsios et al., 2010; Gallant et al., 2005) to form focal adhesion complexes (Kalinina et al., 2008), whereas in non-adherent cells the Vinculin remains diffused in the cytoplasm (Delivopoulos et al., 2015). We found that in retinal cells, both RGD- and YIGSR-based molecules elicited an increase in the focal adhesion spots; the largest effect was found for the YIGSR molecules. In the HEK293 cells, only RGD-based molecules increased the number of FA spots; the largest effect was found for the two cyclic RGD molecules. Our results are in agreement with previous reports that used ECM imitating biomolecules and showed the generation of FA 72 h post-seeding (Ritz et al., 2017; Pallarola et al., 2017; Cavalcanti-Adam et al., 2007; Cavalcanti-adam et al., 2006; Kalinina et al., 2008; Sorribas et al., 2002). Focal adhesions were reported to increase the cell adhesion strength (Gallant et al., 2005; Selhuber-Unkel et al., 2010); therefore, playing an important role in neural tissue engineering (Rao and Winter 2009; Stukel and Willits, 2016; de Luca et al., 2014) and potentially in cell electrode attachment and interaction. Previous studies have highlighted the critical role of integrins and focal adhesion in cellular structures such as cortical (Scuderi et al., 2021) neuron organoids, astrocytes (Rao and Winter, 2009; Kam et al., 2002), or Schwann (Chen et al., 2000) cells. Nevertheless, there is still a lack of information regarding the effect of biomimetic molecules on FA in retinal cells; our study addresses this gap by presenting findings that illuminate the distinctive responses of retinal cells to the various biomolecules.

We also evaluated the effect of the biomolecules on the adhesion-related gene expression levels. We found that the two tested molecules (long-spacer cRGD and short-spacer YIGSR), which were found to be optimal for attracting our cells, increased the expression of the adhesion integrins Intα_V_, Intα_5_, Intβ_1_, and Intβ_3_ as well as the focal adhesion proteins (Vinculin and PTK-2); as expected, the adhesion integrin Intα_IIb_, which is related to platelet activation, was not affected. The magnitude of the effect of RGD on HEK293 gene expression enhancement is similar to previous reports (Ritz et al., 2017; Sobers et al., 2015) in fibroblasts, and was smaller in the retinal cells. Previous work suggests that neural cells natively bind to the YIGSR motif of Laminin through the Intβ_1_ and Intβ_3_ subunits probably via Intα_5_β_1_, Intα_4_β_1_, and Intα_v_β_3,_ respectively (Stukel and Willits, 2016). Indeed, our results present the increment in these integrin subunits, suggesting that cell adhesion is mediated through these integrins. However, more work is needed to validate this by specifically blocking integrins with antibodies and demonstrating the prevention of cell attachment.

In conclusion, in this work we studied the impact of short biomolecules that mimic the ECM adhesion motifs regarding retinal cell attachment to electrode surfaces. We found the YIGSR-type molecules elicited the cellular adhesion mechanism, leading to an increase in cellular density, cell spreading, and FA expression. Future research should study the advantage of combining several biomolecules to further improve cell adhesion and the effect of these coatings on neural electrical activation thresholds.

## Data Availability

The data supporting the current study have not been deposited in a public repository because it was generated by various setups requiring customized analyses software in non-standard format. The data is available from the lead contact (yossi.mandel@biu.ac.il) on request.
