# Supplementary material for: Adhesion of retinal cells to gold surfaces by biomimetic molecules
Source: Front Cell Dev Biol. 2024 Aug 28;12:1438716. doi: 10.3389/fcell.2024.1438716 (PMC11387177; doi:10.3389/fcell.2024.1438716)
Supplement: Supplementary file 1 [file DataSheet1.docx]

**Supplementary Material**

**Adhesion of Retinal Cells to Gold Surfaces by Biomimetic Molecules**

**Gal Shpun^1,2,3^, Amos Markus^2,3^, Nairouz Farah^2,3^, Zeev Zalevsky^1,3^ , Yossi Mandel^2,3,4^**

1. The Alexander Kofkin Faculty of Engineering, Bar Ilan University, 5290002 Ramat Gan, Israel
2. Faculty of Life Sciences, School of Optometry & Visual Science, Bar Ilan University, 5290002 Ramat Gan, Israel
3. Bar Ilan Institute for Nanotechnology & Advanced Materials (BINA), Bar Ilan University, 5290002 Ramat Gan, Israel
4. The Gonda Multidisciplinary Brain Research Centre, Bar-Ilan University, Ramat Gan, Israel

*Correspondence: Yossi Mandel yossi.mandel@biu.ac.il

**Primers for qPCR**

|  |  | **Human** | **Rat** |
| --- | --- | --- | --- |
| **GAPDH** | **FW** | 5'-CACATGGCCTCCAAGGAGTAA-3′^4^ | 5'-CACATGGCCTCCAAGGAGTAA-3′^4^ |
|  | **RV** | 5'-TGAGGGTCTCTCTCTTCCTCTTGT-3′^4^ | 5'-TGAGGGTCTCTCTCTTCCTCTTGT-3′^4^ |
| **ITGA2B** | **FW** | 5'-GCAGATACGGAGCAAGAACA-3′^5^ | 5'-GGCCATGTCTTCAACGGAAT-3′ |
|  | **RV** | 5'-GATGTAGAGCAGGTCGGAGG-3′^5^ | 5'-CAGACCAGGTGCATCCCTTTT-3′ |
| **ITGAV** | **FW** | 5'-AATCTTCCAATTGAGGATATCAC-3′^6^ | 5'-AGGAGGGAATTTGGTGGCAG-3′ |
|  | **RV** | 5'-AAAACAGCCAGTAGCAACAAT-3′^6^ | 5'-TTTGCCGGGTTCCCTTACTC-3′ |
| **ITGA5** | **FW** | 5'-TGCAGTGTGAGGCTGTGTACA-3′^6^ | 5′-TGCTCATCTATCTTCAAACGCTCC-3′ |
|  | **RV** | 5'-GTGGCCACCTGACGCTCT-3′^6^ | 5′-GTGCCTAGTCCCTCCCTACC-3′ |
| **ITGB1** | **FW** | 5'-GAAGGGTTGCCCTCCAGA-3′^6^ | 5′-AAGTGGGACACTACTGGTCCC-3′ |
|  | **RV** | 5'-GCTTGAGCTTCTCTGCTGTT-3′^6^ | 5′-GTCCCACTTGGCATTTTCTCC-3′ |
| **ITGB3** | **FW** | 5'-CCGTGACGAGATTGAGTCA-3′^6^ | 5′-TGCCTGCTCATTTAAGAAGGACT-3′ |
|  | **RV** | 5'-AGGATGGACTTTCCACTAGAA-3′^6^ | 5′-CAGCCGGGGTTTATGACACT-3′ |
| **VCL** | **FW** | 5′-GATGAGCTTGCTCCTCCCAA-3′ | 5′-TCTGTGAGCGAATCCCAACT-3′ |
|  | **RV** | 5′-CATCATGGAGCTGTCTGGCA-3′ | 5′-TCGTCACTGATGTTGGTCCG -3′ |
| **PTK-2** | **FW** | 5′-AATACGGCGATCATACTGGG-3′^7^ | 5′-AAATGCTAGGGCAGACACGG-3′ |
|  | **RV** | 5′-CATGCCTTGCTTTTCGCTGT-3′^7^ | 5′-GCTGCTGGTGGAATGCTAGA-3′ |

**Table Supp-T1. Primers used in Quantitative PCR Analysis of human HEK293 and** rat PRP.

**X-ray photoelectron spectroscopy (XPS) analysis**

In order to evaluate the success of the biomolecule connection to the gold surface, an XPS analysis was performed (Fig. S1) similarly to Yoon et.al (2011)^1^ and Phipps et.al (2006)^2^. Survey and high-resolution spectra were acquired at a pass energy of 80eV and 40eV, respectively. The source power was set to either 75W or 150W. The binding energies of all elements were recalibrated by setting the CC/CH component of the C_1s_ peak at 285eV. Quantitative surface chemical analysis was performed using high-resolution core-level spectra after the removal of the nonlinear Shirley background. The measurements were carried out under UHV conditions, at a base pressure of 5x10^-10^torr (and no higher than 3x10^-9^torr). Examinations were performed on gold-coated mica glass disks (Electron Microscopy Sciences, Hatfield, PA, USA). A sulfuric (S_2p,_ at 162.5eV) peak from the thiol group (S atom in Cysteine) and a nitrogen (N_1s_ at 397eV) peak from the amine group of the peptide, indicating the presence of the RGD molecule. In addition, gold peaks (Au_4s_, Au_4p1/2_, Au_4p3/2_ and Au_4d_ at 463eV, 643eV, 547eV and 335eV, respectively) from the surface, carbon (C_1s_ at 284.8eV, C-C) and oxygen (O_1s_ at 532eV, C=O) peaks from the bonds in the peptide, and contamination of copper from the evaporation process, were also detected^3^. These results are compatible with the reported results of Yoon et.al (2011)^1^.

**Fig. S1. XPS survey spectrum of the modified gold surface revealing the presence of RGD.** In the inserts, zoom-in on the N_1s_ and S_2p_ peaks show the presence of the amin (NH_3_) and thiol (SH) groups from the peptide on the gold surface, respectively.


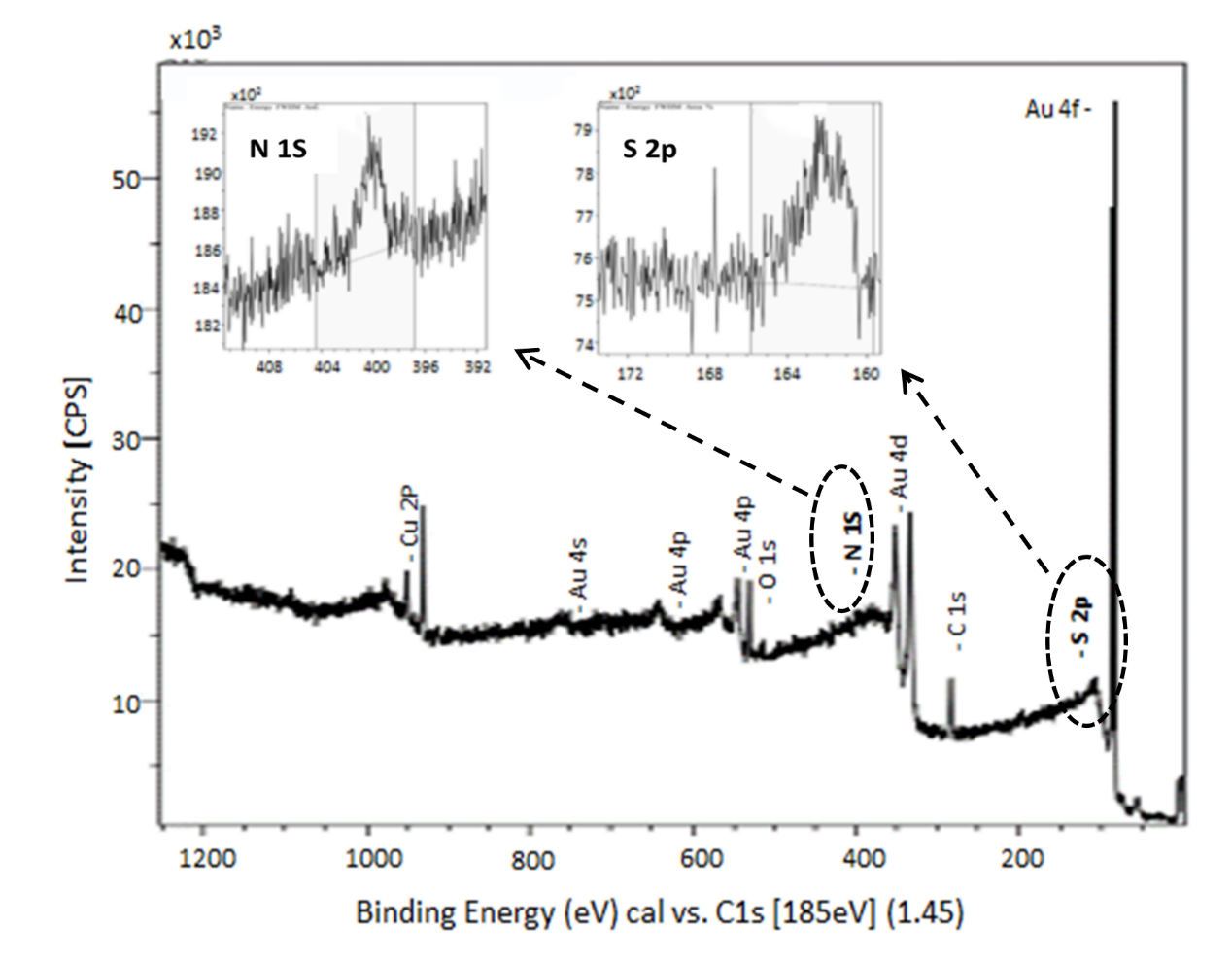


**Biomolecule effects on Cell density**

| **Coating 1** | **Coating 2** | **p-value** |
| --- | --- | --- |
| **DDW** | **GG-RGD** | **0.001** |
| DDW | GG-c(RGD) | 0.999 |
| **DDW** | **Poly-Pro-c(RGD)** | **0.001** |
| DDW | GG-YIGSR | 0.397 |
| DDW | Poly-Pro-YIGSR | 0.415 |
| **GG-RGD** | **GG-c(RGD)** | **0.001** |
| GG-RGD | Poly-Pro-c(RGD) | 0.443 |
| **GG-RGD** | **GG-YIGSR** | **0.001** |
| **GG-RGD** | **Poly-Pro-YIGSR** | **0.001** |
| **GG-c(RGD)** | **Poly-Pro-c(RGD)** | **0.001** |
| GG-c(RGD) | GG-YIGSR | 0.655 |
| GG-c(RGD) | Poly-Pro-YIGSR | 0.683 |
| **Poly-Pro-c(RGD)** | **GG-YIGSR** | **0.001** |
| **Poly-Pro-c(RGD)** | **Poly-Pro-YIGSR** | **0.001** |
| GG-YIGSR | Poly-Pro-YIGSR | 0.999 |

A detailed comparison of the effect of the various biomolecules on cell density for both cell types using T-test comparison between all biomolecule permutations (Fig. 6a-b in the main text) for a concentration of 1/3 [mg/ml] is presented in Supp T2-3.

| **Coating 1** | **Coating 2** | **p-value** |
| --- | --- | --- |
| DDW | GG-RGD | 0.995 |
| DDW | GG-c(RGD) | 0.433 |
| DDW | Poly-Pro-c(RGD) | 0.999 |
| **DDW** | **GG-YIGSR** | **0.012** |
| **DDW** | **Poly-Pro-YIGSR** | **0.002** |
| GG-RGD | GG-c(RGD) | 0.845 |
| GG-RGD | Poly-Pro-c(RGD) | 0.999 |
| **GG-RGD** | **GG-YIGSR** | **0.012** |
| **GG-RGD** | **Poly-Pro-YIGSR** | **0.002** |
| GG-c(RGD) | Poly-Pro-c(RGD) | 0.680 |
| **GG-c(RGD)** | **GG-YIGSR** | **0.001** |
| **GG-c(RGD)** | **Poly-Pro-YIGSR** | **0.001** |
| **Poly-Pro-c(RGD)** | **GG-YIGSR** | **0.012** |
| **Poly-Pro-c(RGD)** | **Poly-Pro-YIGSR** | **0.002** |
| GG-YIGSR | Poly-Pro-YIGSR | 0.980 |

**Supp-T2 T-test for HEK293 cell density.** The pairs of biomolecules with a significant difference, indicated by p-values < 0.05, are shown in bold.

**Supp-T3 T-test for retinal cell density.** The pairs of biomolecules with a significant difference, indicated by p-values < 0.05, are shown in bold.

**Cell spreading following various bio-molecules surface treatment**

Representative images of cell surface area for HEK293 cells and retinal cells are presented in Fig. S2 and S3, respectively. Both cell types were seeded on a coated gold surface with a biomolecule concentration of 1/6 mg/ml and incubated for 72h followed by fixation and staining.


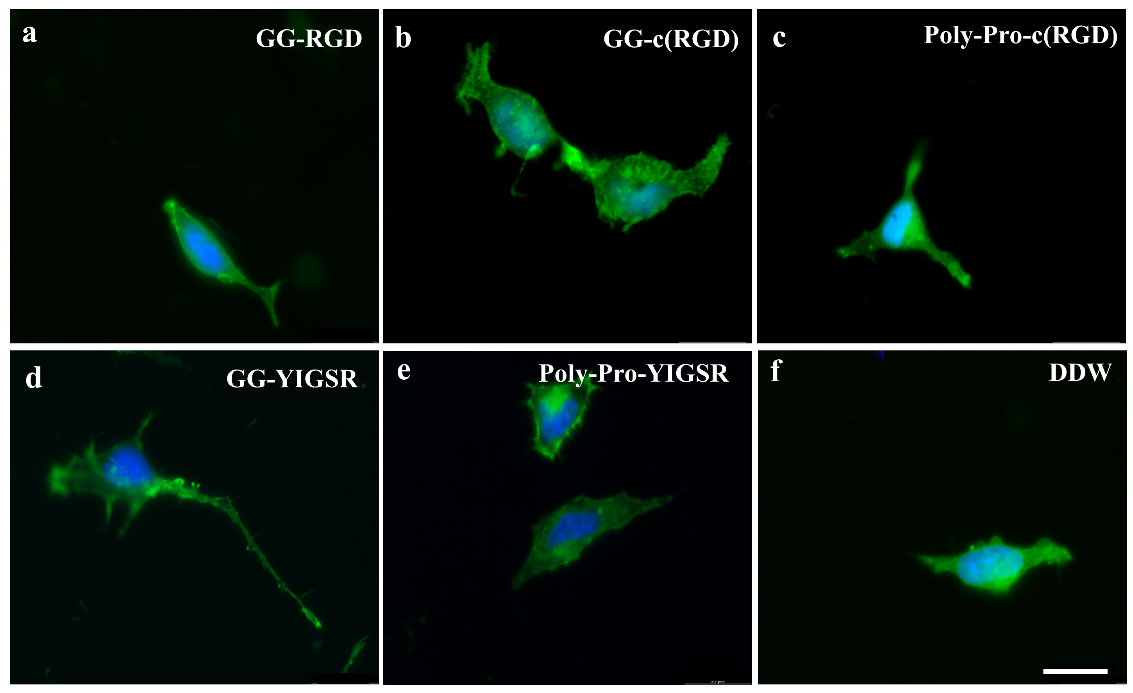


**Fig. S2. Confocal images of the coating biomolecule effect on HEK293 cells surface area of.** **(a)** Short linear RGD, **(b)** short cyclo-RGD, **(c)** long cyclo-RGD, **(d)** short YIGSR, **(e)** long YIGSR. **(f)** DDW only. Blue Hoechst, Green cytoplasmic genetic encoded GFP. Scale bar 25µm

**Fig S3. Confocal images of the coating biomolecule effect on rat dissociated retinal cells surface area.** **(a)** Short linear RGD, **(b)** short cyclo-RGD, **(c)** long cyclo-RGD, **(d)** short YIGSR, **(e)** long YIGSR. **(f)** DDW only. Blue Hoechst, Green cytoplasmic staining ViaFluor 488. Scale bar 20µm.


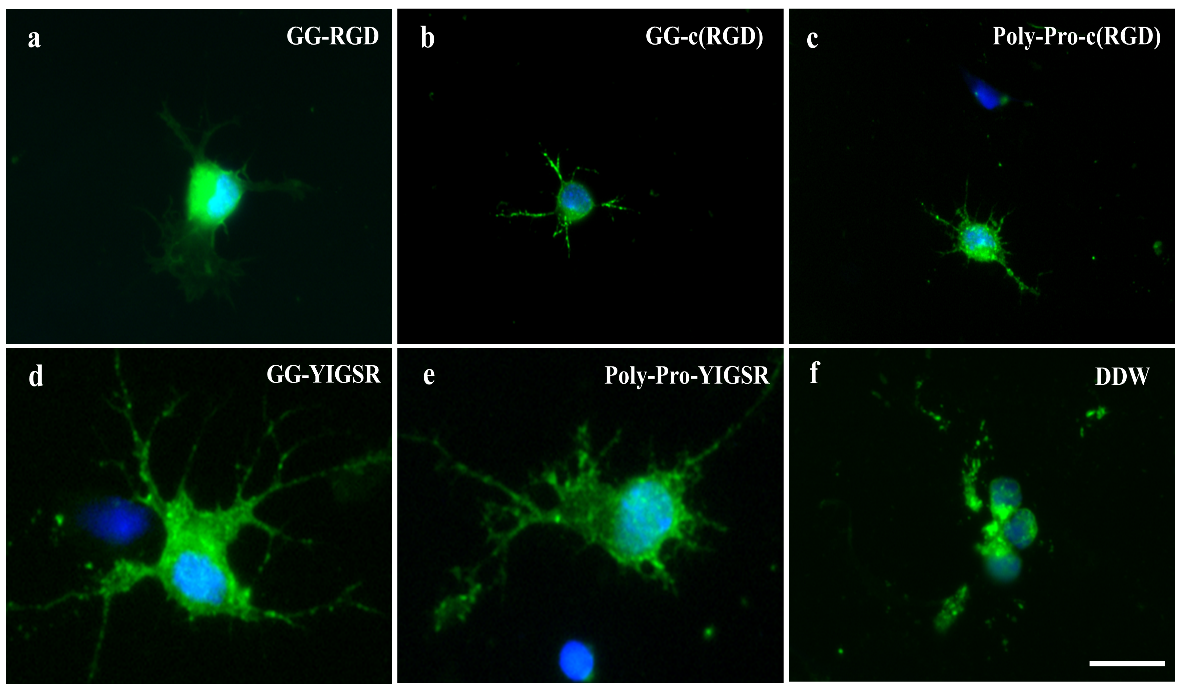


**The effect of biomolecules surface treatment on cells spreading**

A detailed comparison of the effect of the various biomolecules on cell spreading for both cell types using T-test comparison between all biomolecule permutations is presented in Supp T4- SuppT5.

| **Coating 1** | **Coating 2** | **p-value** |
| --- | --- | --- |
| **DDW** | **GG-RGD** | **0.001** |
| **DDW** | **GG-c(RGD)** | **0.001** |
| **DDW** | **Poly-Pro-c(RGD)** | **0.001** |
| **DDW** | **GG-YIGSR** | **0.001** |
| DDW | Poly-Pro-YIGSR | 0.147 |
| GG-RGD | GG-c(RGD) | 0.644 |
| GG-RGD | Poly-Pro-c(RGD) | 0.891 |
| GG-RGD | GG-YIGSR | 0.996 |
| GG-RGD | Poly-Pro-YIGSR | 0.348 |
| GG-c(RGD) | Poly-Pro-c(RGD) | 0.997 |
| GG-c(RGD) | GG-YIGSR | 0.898 |
| **GG-c(RGD)** | **Poly-Pro-YIGSR** | **0.011** |
| Poly-Pro-c(RGD) | GG-YIGSR | 0.992 |
| **Poly-Pro-c(RGD)** | **Poly-Pro-YIGSR** | **0.039** |
| GG-YIGSR | Poly-Pro-YIGSR | 0.145 |

| **Coating 1** | **Coating 2** | **p-value** |
| --- | --- | --- |
| DDW | GG-RGD | 0.776 |
| DDW | GG-c(RGD) | 0.967 |
| DDW | Poly-Pro-c(RGD) | 0.999 |
| **DDW** | **GG-YIGSR** | **0.001** |
| **DDW** | **Poly-Pro-YIGSR** | **0.001** |
| GG-RGD | GG-c(RGD) | 0.539 |
| GG-RGD | Poly-Pro-c(RGD) | 0.859 |
| **GG-RGD** | **GG-YIGSR** | **0.001** |
| **GG-RGD** | **Poly-Pro-YIGSR** | **0.001** |
| GG-c(RGD) | Poly-Pro-c(RGD) | 0.999 |
| **GG-c(RGD)** | **GG-YIGSR** | **0.001** |
| **GG-c(RGD)** | **Poly-Pro-YIGSR** | **0.001** |
| **Poly-Pro-c(RGD)** | **GG-YIGSR** | **0.001** |
| **Poly-Pro-c(RGD)** | **Poly-Pro-YIGSR** | **0.001** |
| GG-YIGSR | Poly-Pro-YIGSR | 0.990 |

**The effect of biomolecule surface treatment on focal adhesions**

**Supp-T4 T-test for HEK293 cells’ surface area.** The pairs of biomolecules with a significant difference, indicated by p-values < 0.05, are shown in bold.

**Supp-T5 T-test for retinal cells’ surface area.** The pairs of biomolecules with a significant difference, indicated by p-values < 0.05, are shown in bold.

| **Coating 1** | **Coating 2** | **p-value** |
| --- | --- | --- |
| DDW | GG-RGD | **0.047** |
| DDW | GG-c(RGD) | **0.000** |
| DDW | Poly-Pro-c(RGD) | **0.000** |
| DDW | GG-YIGSR | 0.954 |
| DDW | Poly-Pro-YIGSR | 1.000 |
| GG-RGD | GG-c(RGD) | 0.901 |
| GG-RGD | Poly-Pro-c(RGD) | 0.903 |
| GG-RGD | GG-YIGSR | 0.282 |
| GG-RGD | Poly-Pro-YIGSR | 0.035 |
| GG-c(RGD) | Poly-Pro-c(RGD) | 1.000 |
| GG-c(RGD) | GG-YIGSR | **0.005** |
| GG-c(RGD) | Poly-Pro-YIGSR | **0.000** |
| Poly-Pro-c(RGD) | GG-YIGSR | **0.006** |
| Poly-Pro-c(RGD) | Poly-Pro-YIGSR | **0.000** |
| GG-YIGSR | Poly-Pro-YIGSR | 0.911 |

Aiming to compare the results of the focal adhesion complexes formation (Fig 10 a-b in the main text), the T-test between all the investigated biomolecule permutations was calculated and presented below in Supp-T3 and Supp-T4, respectively.

**Supp-T6 T-test for HEK293 cells’ focal adhesion.** The pairs of biomolecules with a significant difference, indicated by p-values < 0.05, are shown in bold.

| **Coating 1** | **Coating 2** | **p-value** |
| --- | --- | --- |
| DDW | GG-RGD | **0.003** |
| DDW | GG-c(RGD) | **0.044** |
| DDW | Poly-Pro-c(RGD) | **0.007** |
| DDW | GG-YIGSR | **0.002** |
| DDW | Poly-Pro-YIGSR | **0.011** |
| GG-RGD | GG-c(RGD) | 0.772 |
| GG-RGD | Poly-Pro-c(RGD) | 0.928 |
| GG-RGD | GG-YIGSR | **0.050** |
| GG-RGD | Poly-Pro-YIGSR | 0.807 |
| GG-c(RGD) | Poly-Pro-c(RGD) | 0.992 |
| GG-c(RGD) | GG-YIGSR | 0.567 |
| GG-c(RGD) | Poly-Pro-YIGSR | 1.000 |
| Poly-Pro-c(RGD) | GG-YIGSR | 0.740 |
| Poly-Pro-c(RGD) | Poly-Pro-YIGSR | 0.999 |
| GG-YIGSR | Poly-Pro-YIGSR | 0.566 |

**Supp-T7 T-test for retinal cells’ focal adhesion.** The pairs of biomolecules with a significant difference, indicated by p-values < 0.05, are shown in bold.

**References**

1. Yoon, S. H. & Mofrad, M. R. K. Cell adhesion and detachment on gold surfaces modified with a thiol-functionalized RGD peptide. *Biomaterials* **32**, 7286–7296 (2011).

2. Phipps, Crystal M. Surface modification of titanium nitride to increase cellular adhesion, University of Southern California. ProQuest Dissertations & Theses, 2006. 1437854.

3. Moulder, J. F., Stickle, W. F., Sobol, P. E. ’ & Bomben, K. D. *Handbook of X-Ray Photoelectron Spectroscopy*. (Perkin-Elmer Corporation Physical Electronics Division 6509 Flying Cloud Drive, Eden Prairie, MN 55344, USA, 1992).

4. Schick, R., Farah, N., Markus, A., Korngreen, A. & Mandel, Y. Electrophysiologic characterization of developing human embryonic stem cell-derived photoreceptor precursors. *Invest Ophthalmol Vis Sci* **61**, (2020).

5. Mohit Trikha *et al.* Human Prostate Carcinoma Cells Express Functional αIIbβ3 integrin. *Cancer Res* **56**, 5071–5078 (1996).

6. Dingemans, A. M. C. *et al.* Integrin expression profiling identifies integrin alpha5 and beta1 as prognostic factors in early stage non-small cell lung cancer. *Mol Cancer* **9**, (2010).

7. Mu, L., Chen, W., Ma, Y. & Zheng, W. Expression of focal adhesion kinase in the eutopic endometrium of women with adenomyosis varies with dysmenorrhea and pelvic pain. *Exp Ther Med* **10**, 1903–1907 (2015).
